# The Broad-Spectrum Antiviral Protein ZAP Restricts Human Retrotransposition

**DOI:** 10.1371/journal.pgen.1005252

**Published:** 2015-05-22

**Authors:** John L. Goodier, Gavin C. Pereira, Ling E. Cheung, Rebecca J. Rose, Haig H. Kazazian

**Affiliations:** McKusick-Nathans Institute of Genetic Medicine, Johns Hopkins University School of Medicine, Baltimore, Maryland, United States of America; Fred Hutchinson Cancer Research Center, UNITED STATES

## Abstract

Intrinsic immunity describes the set of recently discovered but poorly understood cellular mechanisms that specifically target viral pathogens. Their discovery derives in large part from intensive studies of HIV and SIV that revealed restriction factors acting at various stages of the retroviral life cycle. Recent studies indicate that some factors restrict both retroviruses and retrotransposons but surprisingly in ways that may differ. We screened known interferon-stimulated antiviral proteins previously untested for their effects on cell culture retrotransposition. Several factors, including BST2, ISG20, MAVS, MX2, and ZAP, showed strong L1 inhibition. We focused on ZAP (PARP13/ZC3HAV1), a zinc-finger protein that targets viruses of several families, including Retroviridae, Tiloviridae, and Togaviridae, and show that ZAP expression also strongly restricts retrotransposition in cell culture through loss of L1 RNA and ribonucleoprotein particle integrity. Association of ZAP with the L1 ribonucleoprotein particle is supported by co-immunoprecipitation and co-localization with ORF1p in cytoplasmic stress granules. We also used mass spectrometry to determine the protein components of the ZAP interactome, and identified many proteins that directly interact and colocalize with ZAP, including MOV10, an RNA helicase previously shown to suppress retrotransposons. The detection of a chaperonin complex, RNA degradation proteins, helicases, post-translational modifiers, and components of chromatin modifying complexes suggest mechanisms of ZAP anti-retroelement activity that function in the cytoplasm and perhaps also in the nucleus. The association of the ZAP ribonucleoprotein particle with many interferon-stimulated gene products indicates it may be a key player in the interferon response.

## Author Summary

Retrotransposons are mobile DNA elements that duplicate themselves by a "copy and paste" mechanism using an RNA intermediate. They are insertional mutagens that have had profound effects on genome evolution, fostering DNA deletions, insertions and rearrangements, and altering gene expression. LINE-1 retrotransposons occupy 17% of human DNA, although it is believed that only about 100 remain competent for retrotransposition in any individual. The cell has evolved defenses restricting retrotransposition, involving in some cases interferon-stimulated genes (ISGs) that are part of the innate immune system that protects the cell from viral infections. We screened a panel of ISGs and found several to strongly limit retrotransposition in a cell culture assay. Our investigations increase understanding of how ZAP, an important restriction factor against positive- and negative-strand RNA and some DNA viruses, also interacts with human retrotransposons to prevent genome mutation. Microscopy and immunoprecipitation show a close association of ZAP protein with the L1 ribonucleoprotein particle, as well as MOV10, an RNA helicase that also inhibits retrotransposons. A detailed examination of the ZAP protein interactome reveals many other ISGs that directly bind ZAP, and suggests new directions for exploring the mechanisms of ZAP-mediated anti-retroelement activity.

## Introduction

Host restriction factor proteins are part of the intrinsic immune system of the cell, forming an early line of defense against viral infection. Intrinsic immunity is triggered when viral RNAs are recognized by pattern-recognition receptors, such as Toll-like and retinoic acid-inducible gene (RIG-I)-like receptor family members, causing activation of an effector protein (for example, IRF3) and the expression of interferon (IFN) and hundreds of IFN-stimulated genes (ISGs).

Many viral restriction factors are ISGs that function by diverse mechanisms against a wide range of viral pathogens. For example, Myxovirus (influenza virus) resistance 1, interferon-inducible protein p78 (mouse) (MX1, also known as MXA)) and MX2 (MXB) are closely related members of the IFN-induced dynamin family of large GTPases. MX1 is a broad-spectrum inhibitor of many RNA and DNA viruses (reviewed in [1]). IFN-induced transmembrane protein family members (IFITM1/2/3) are also potent inhibitors of a range of viruses including HIV-1, although their mechanisms of action are unclear ([2]; reviewed in [3]). BST2 (Tetherin) is a type II transmembrane glycoprotein capable of trapping enveloped virions at the cell surface (reviewed in [4]). RSAD2 (Viperin) is an endoplasmic reticulum-associated protein that inhibits many RNA and DNA viruses at multiple stages of the viral life cycle, and which may be involved in innate immune signaling (reviewed in [5]). RNA helicases and IFIH1 interact with Mitochondrial antiviral signaling protein (MAVS), a mitochondrial outer membrane protein, activating formation of the MAVS signalosome and upregulation of NF-κB and IRF3 signaling pathways [6]. ISG20 is a 3'-5' exoribonuclease that inhibits single-strand RNA viruses including HIV-1 [7]. The transcriptional regulator TRIM28 (KAP1) also limits HIV integration by binding acetylated integrase and inducing its deacetylation by recruiting HDAC1 [8].

While the cell can be infected by a wide variety of viruses, unrestricted activity of endogenous retroelements also poses a threat to genome integrity and cell function. Long-terminal repeat (LTR) retrotransposons include the human endogenous retroviruses (HERVs) that comprise 8% of the human genome, although no HERVs capable of replication have been identified. However, increased HERV expression has been implicated in multiple sclerosis, lupus, amyotrophic lateral sclerosis, and autoimmune rheumatic disease, although whether the increase is the cause or effect of disease remains to be determined ([9]; reviewed in [10–12]).

Non-LTR retrotransposons comprise at least one-third of the human genome and remain an ongoing cause of disease [13,14]. LINE-1s (L1s) are the only active autonomous mobile DNA remaining in humans, and among ∼500,000 copies at least 100 remain potentially active for retrotransposition in any human individual [15,16]. L1s have also been responsible for the genomic insertion *in trans* of thousands of processed pseudogenes and a million SINEs (Alus and SVAs). The current residual activity of human retrotransposons is the background that escapes a variety of mechanisms that have evolved to limit replication of mobile DNA.

Phylogenetic analyses suggest that eukaryote non-LTR retrotransposons predate LTR retrotransposons, which in turn gave rise to the retroviruses through the acquisition of an envelope gene [17–19]. The ancient origin and interrelatedness of the major classes of retroelements predicts they will be subject to some of the same host restriction factors. On the other hand, significant differences between their modes of replication suggest that non-LTR retrotransposons and retroviruses could be affected by the same restriction factors in divergent ways. For example, Apolipoprotein B mRNA-editing enzyme, catalytic polypeptide-like 3 (APOBEC3) family members, first shown to inhibit HIV by hypermutation of minus-strand DNA, were then found to potently inhibit retrotransposition but without obvious hypermutation, suggesting a unique pathway (reviewed in [20,21]). Recently, it was proposed that APOBEC3A inhibits L1 retrotransposition by deaminating transiently exposed single-strand genomic DNA that flanks the site of L1 integration [22]. The Aicardi-Goutières syndrome (AGS)-related anti-retroviral protein SAM domain and HD domain 1 protein (SAMHD1) inhibits retroviruses in non-dividing myeloid cells and resting CD4+ T cells by depleting dNTP levels. However, SAMHD1 also limits non-LTR retrotransposition in dividing cells, suggesting an inhibitory mechanism different from nucleotide depletion [23].

Other innate restriction factors also suppress retrotransposons. LINE-1 activity is upregulated in cells deficient for TREX1, a gene associated with systemic lupus erythematosus (SLE) and, like SAMHD1, with AGS [24]. RNaseL, a member of the IFN antiviral response to dsRNA, likely restricts retrotransposons in cell culture by cleavage of their mRNAs [25]. Finally, the RNA helicase MOV10, previously reported to affect replication of several RNA viruses, also limits activity of all human retrotransposons [26–28]. MOV10 may target retroelement complexes for degradation, possibly by RNA-induced silencing complexes (RISC). Clearly, studying factors that restrict both retrotransposons and retroviruses can inform both fields.

Therefore, we overexpressed a panel of antiviral ISGs and assayed for their previously untested effects on LINE-1 retrotransposition in a cell culture assay. Several of these factors strongly inhibited retrotransposition of an L1 reporter construct. We report that the zinc finger antiviral protein ZAP (also called Zinc finger CCCH-type, antiviral 1, ZC3HAV1, and Poly (ADP-ribose) polymerase 13, PARP13) is a potent inhibitor of not only viruses but also retrotransposons. ZAP targets positive- and negative-strand RNA viruses of several families, including Retroviridae (HIV-1, MoLV, and XMRV), Filoviridae (Ebola and Marburg), Togaviridae (alpha-, sindbis, Semliki Forest, and Ross river viruses), and Hepadnaviridae (hepatitis B) [29], and has been shown to inhibit the double-stranded DNA murine gammaherpesvirus [30]. However, restriction is not universal: ZAP fails to inhibit vesicular stomatitis, poliovirus, yellow fever, and herpes simplex I viruses [31]. The presence of orthologs in fish, birds and reptiles suggests that ZAP (like MOV10) is ancient in origin [32,33].

L1 expresses a 6-kb bicistronic RNA that encodes a 40 kD RNA-binding protein (ORF1p) of essential but uncertain function for retrotransposition, and a 150 kD ORF2 protein with endonuclease and reverse transcriptase (RT) activities. In the cytoplasm, ORF1p and ORF2p preferentially bind their own encoding RNA *in cis* to form a functional ribonucleoprotein particle (RNP) [34,35]. We show that ZAP strongly restricts retrotransposition in cell culture with loss of L1 RNA and RNP integrity. Association of ZAP with the L1 RNP is supported by co-IP and co-localization with ORF1p in cytoplasmic granules. Mass spectrometry (MS) analyses of the ZAP proteome revealed proteins that bind ZAP, including MOV10, and suggest possible mechanisms of ZAP-mediated retroelement restriction.

## Results

### ISGs limit LINE1 retrotransposition in a cell culture assay

We asked if increased interferon limits L1 activity in a cell culture assay for retrotransposition. In this assay, an enhanced green fluorescent protein (EGFP) reporter gene cassette, interrupted by a backwards intron and inserted in opposite transcriptional orientation into the 3' UTR of L1-RP (a highly active L1 [36]), is expressed only when the L1 transcript is spliced, reverse-transcribed, its cDNA inserted in the genome, and the EGFP reporter gene expressed from its own promoter [37,38]. The full-length L1 and reporter cassette are cloned in a modified version of pCEP4 vector (Invitrogen) lacking a cytomegalovirus (CMV) promoter. Therefore, expression of the L1 is driven by its own 5' UTR.

**Fig 1:**
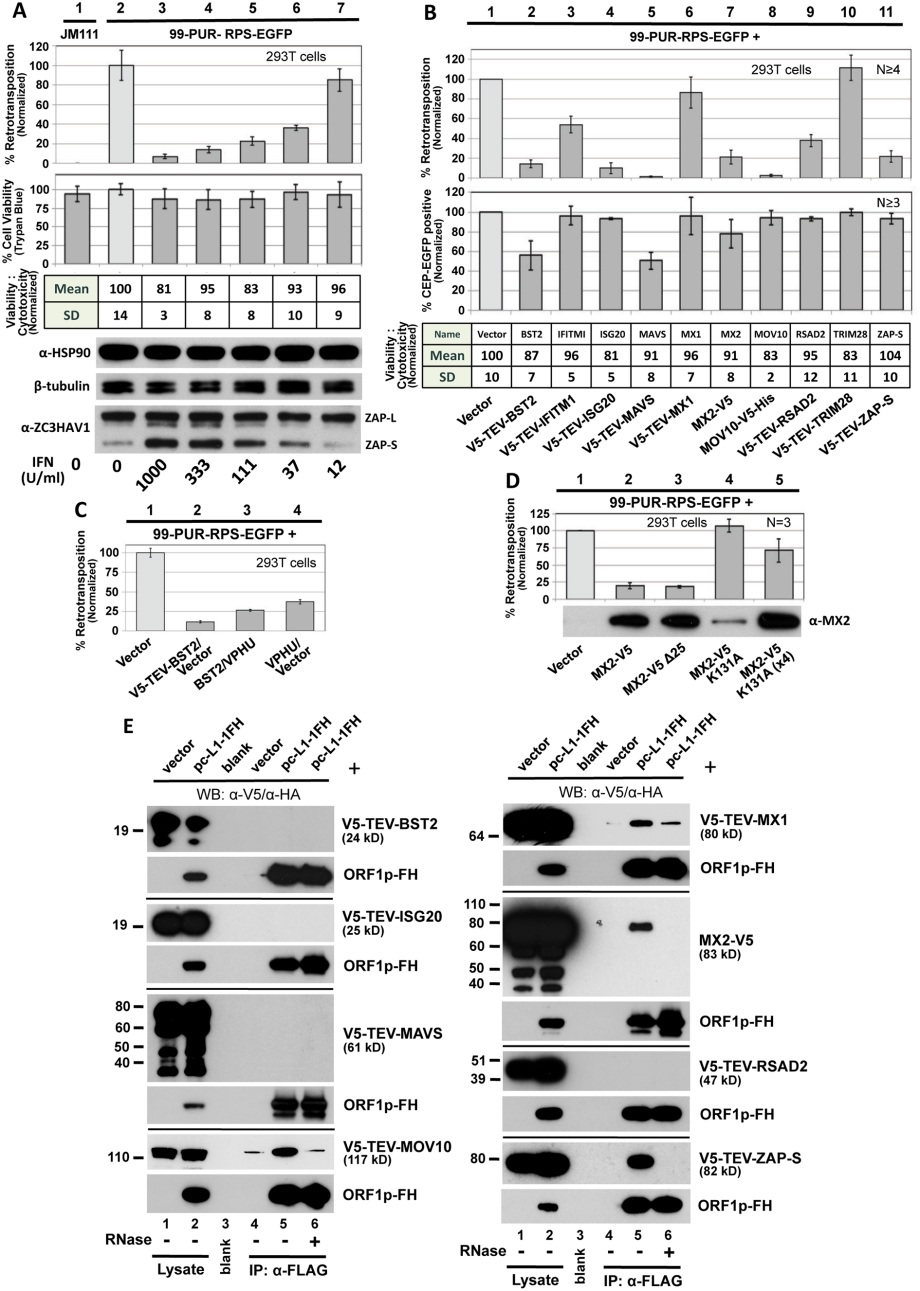
Selected ISG proteins strongly inhibit L1 retrotransposition in 293T cells. (A) Type I interferon inhibits cell culture retrotransposition in a dose-dependent manner. Universal Type I Interferon (PBL Assay Science) at increasingly dilute concentrations was applied at the time of transfection with 99-PUR-RPS-EGFP and replenished once during the course of the experiment. Flow cytometry was performed on Day 5. Four replicate wells were analyzed for each concentration tested. Normalized percentages of retrotransposition-positive cells are shown (top bar chart). To assess possible cytotoxicity caused by interferon, cells were 1) stained on day 5 with trypan blue and counted using a Cellometer Auto T4 Cell Viability Counter (Nexcelom) (lower bar chart), and 2) assayed with the MultiTox-Fluor Multiplex Cytotoxicity assay (table). Western blotting (below) shows that treatment with interferon did not significantly alter expression of endogenous heat shock protein 90 (HSP90) or β-tubulin-2, but strongly induced expression of the short but not the long isoform of ZAP. For protein preparations, an equal volume of cells was removed from each of the four test wells prior to FACs analysis and pooled. JM111: construct 99-PUR-JM111-EGFP, having an ORF1p double-point mutant, was used as a negative control for retrotransposition. (B) Top panel: 99-PUR-RPS-EGFP was cotransfected with empty vector (pcDNA3 or pcDNA6 myc/his B) or test constructs expressing V5-tagged proteins. Five days later, the numbers of EGFP-positive cells were determined by flow cytometry. Each construct pair was tested in quadruplicate wells for each of 4 to 6 independent experiments. Results are normalized to empty vector control (pale shaded bar). Middle panel: To control for off-target effects, test constructs were cotransfected with CEP-EGFP, a plasmid that constitutively expresses EGFP. Four days later, cells were assayed for gain or loss of fluorescent cells. Percentages of fluorescent cells are normalized to the empty vector control. Lower table: Results of MultiTox-Fluor Multiplex Cytotoxicity assay (Promega) for potential cell toxicity caused by overexpression of test proteins. Test constructs were transfected in 293T cells in 96-well plates and assayed at 3 days. The bar chart shows ratios of live to dead cell readings normalized to empty vector control. Results of a single experiment are shown. (C) Inhibition of 99-PUR-RPS-EGFP retrotransposition by BST2 (tetherin) is partially relieved by cotransfection of a codon-optimized version of HIV-1-encoded Vpu. (D) MX2 with a K131A mutation has diminished ability to inhibit retrotransposition, but loss of its N-terminal nuclear localization signal (MX2-V5 Δ25, lane 3) maintains inhibition. Amounts of the K131A mutant plasmid cotransfected were 0.5 μg (lane 4) and 2 μg (4X; lane 5). Proteins were sampled at 5 days post-transfection and detected by α-MX2 antibody. (E) Ectopically expressed V5-tagged MOV10, MX1, MX2, and ZAP proteins associate in an RNA-dependent manner with L1 complexes expressed from pc-L1-1FH in 293T cells. Input lysate, lanes 1,2; IP on α-FLAG affinity gel, lanes 4–6: Western blotting (WB) with α-V5 antibody to detect test proteins (upper panel for each test protein); WB with α-HA antibody to detect ORF1p-FH (lower panels). IP reactions were in the absence (lanes 1–5) or presence (lane 6) of 50 μg/ml RNase. Molecular weight indicated for each protein includes the epitope tag. Exposure times for the different panels vary.

We transfected HEK 293T cells with the L1-EGFP reporter (99-PUR-RPS-EGFP) in the presence of increasing amounts of Universal Type I Interferon (IFN) alpha, and at 5 days post-transfection assayed for fluorescent (i.e. retrotransposition-positive) cells by flow cytometry. Addition of IFN reduced cell culture retrotransposition in a dose-dependent manner. At 1000 U IFN/ml, retrotransposition was over 90 percent less than non-treated controls, with a loss of cell viability of less than 20 percent as determined by trypan blue staining and the MultiTox-Fluor Multiplex Cytotoxicity Assay (Promega) (Fig 1A, compare lanes 2 and 3).

Several interferon-inducible proteins are known to restrict not only viruses but also endogenous retroelements. To extend the list of these proteins, we next screened a panel of ISGs and restriction factors with known antiviral activities for effects of their expression on L1 retrotransposition (Fig 1B, upper bar chart). To reveal differences of transfection efficiency, cytotoxicity or reporter gene expression caused by the test proteins, we followed the approach of Wei et al. [39] and in conjunction with the retrotransposition assays transfected CEP-EGFP, a vector that constitutively expresses EGFP, together with empty vector or test constructs. After 4 days we performed flow cytometry to assay for loss of green cells (Fig 1B, lower bar chart). It should be noted that the CEP-EGFP assay is a snapshot of GFP fluorescence at a single time-point, while the retrotransposition assay measures accumulated EGFP insertions that occur over 5 days. Therefore, the MultiTox-Fluor Multiplex Cytotoxicity Assay was also used as a direct assay for cell toxicity (assessed at Day 3 post-transfection). This dual-detection assay generates a ratio of live to dead cell readings, thereby normalizing for cell number (Fig 1B, table).

293T cells were cotransfected with 99-PUR-RPS-EGFP and empty vector or tagged ISG cDNA constructs. With the exception of MOV10, none of the proteins shown in Fig 1B had previously been tested for effects on cell culture retrotransposition. Six factors, MOV10 (as previously reported [26–28]), BST2, ISG20, MAVS, MX2, and ZAP, reduced plasmid-directed LINE1 retrotransposition in 293T cells by over 75 percent (Fig 1B). While minimal toxicity was detected by the MultiTox-Fluor assay for all proteins, BST2 and MAVS reduced CEP-EGFP vector expression 44 and 50 percent, respectively. However, loss of CEP-EGFP signal only partly explains the strong inhibition of cell culture retrotransposition observed for these proteins (86 and over 98 percent, respectively).

To assess specificity of BST2-induced reduction of retrotransposition, we coexpressed Vphu, a codon-optimized version of the HIV-1-encoded BST2 antagonist Vpu [40]. Vphu restored L1 retrotransposition over 2-fold (11 to 26 percent), an amount limited by the fact that overexpression of Vphu itself reduced retrotransposition to 37 percent (Fig 1C).

We also tested MX2 mutant proteins for their activity in the cell culture assay (Fig 1D). The N-terminal 25 amino acids of MX2 encode a nuclear envelope targeting domain essential for HIV-1 inhibition. Conversely, while MX2 mutated at K131A fails to bind GTP, it still inhibits HIV-1 as if wild-type [41–44]. Contrary to the results for HIV-1, deleting the first 25 residues of our MX2 construct failed to block its inhibition of retrotransposition, but the K131A mutant lost ability to restrict L1s. Because the K131A mutant is expressed at a lower level than the wild-type protein (also noted by [45]), we increased the amount of mutant MX2 plasmid transfected fourfold but still failed to restore inhibition of retrotransposition to wild-type levels. Thus, the mechanisms by which MX2 restricts retroviruses and retrotransposons appear to differ in some aspects. Unfortunately, endogenous MX2 is expressed at detectable levels in none of the cell lines we use for our retrotransposition assay (293T, HeLa, or human embryonal carcinoma 2102Ep cells) and is induced by IFN in HeLa cells only (S1 Fig). Consequently, we could not assay endogenous MX2 for effect on retrotransposition, and we pursued MX2-related experiments no further.

Finally, we tested for interaction of IGS factors with the L1 RNP (Fig 1E). Expression of pc-L1-1FH, a construct containing L1-RP with a tandem FLAG-HA tag fused to the C-terminus of its ORF1, permits immunoprecipitation (IP) of basal L1 RNP complexes [46]. pc-L1-1FH and V5 epitope-tagged ISG proteins were coexpressed in 293T cells, followed by their co-IP on α-FLAG agarose. TRIM28 and IFITM1 bound non-specifically to the affinity agarose, and their associations with L1 were inconclusive. MOV10 (as previously reported [27]), MX1, MX2, and the short isoform of ZAP (ZAP-S/PARP13.2) all co-immunoprecipitated with the L1 RNP. These interactions were lost with RNase treatment. We have not confirmed that these proteins directly bind L1 RNA. Rather, proteins might bind non-L1 RNAs or other multi-protein complexes captured within the L1 RNP. Moreover, an unknown amount of tagged ORF1p along with its bound partners, free in solution and not part of retrotransposition-competent RNPs, will have co-purified within immunoprecipitates. Only functional analyses of restriction factors associated with the L1 RNP will ascertain their relevance to L1 biology.

Thus, we have expanded the growing list of IFN-stimulated cellular restriction factors that inhibit human retrotransposition. We decided to focus on the zinc finger protein ZAP for further investigation.

### The ZAP zinc finger domain is sufficient for restriction of retrotransposition

**Fig 2:**
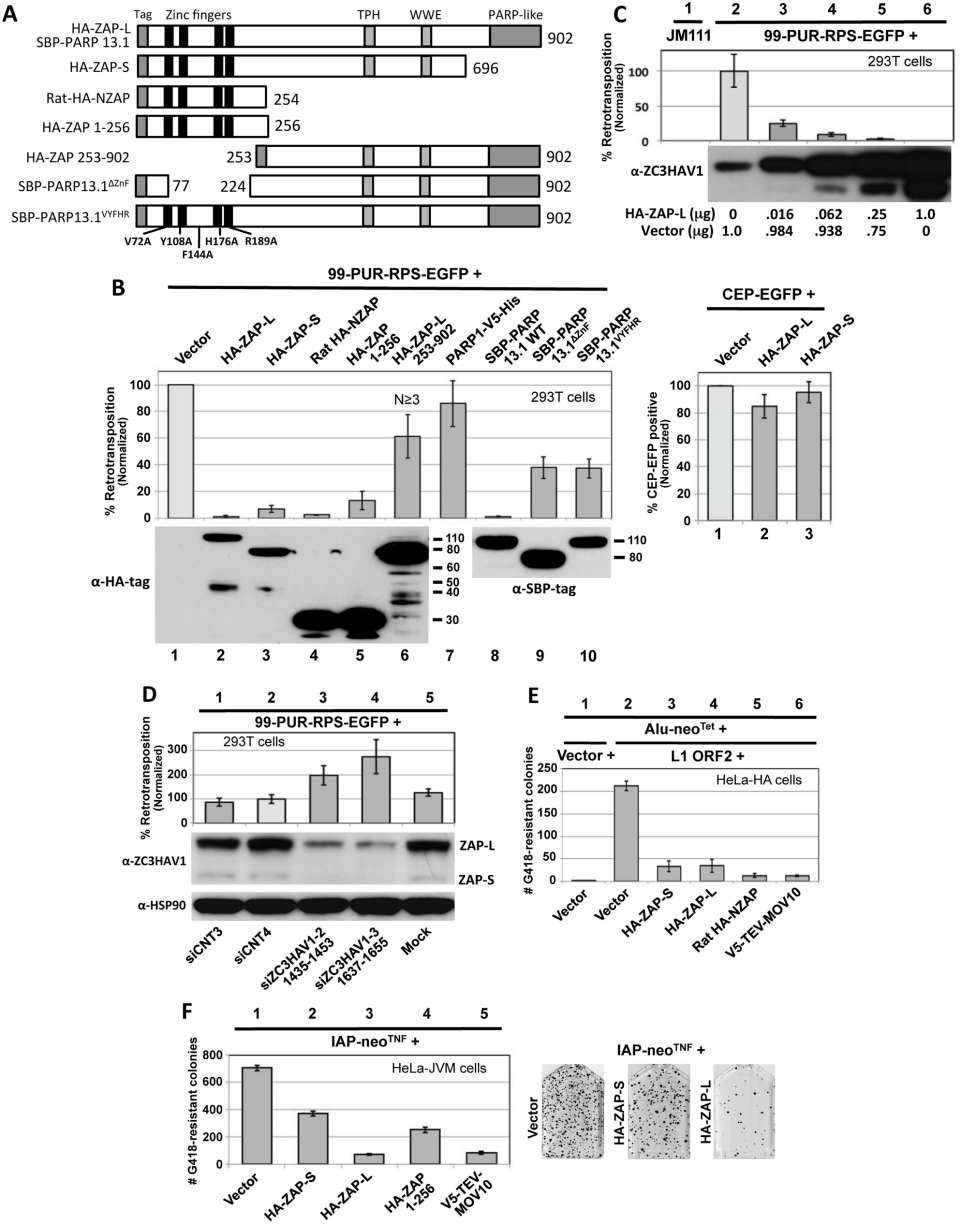
Evidence from cell culture assays that ZAP inhibits retrotransposition. (A) The structure of ZAP constructs used in the retrotransposition assays. Known protein domains are indicated. (B) Left upper panel, right: 99-PUR-RPS-EGFP was cotransfected in 293T cells with empty vector or constructs expressing tagged ZAP constructs, and 5 days later the percentages of EGFP-positive cells were determined by flow cytometry. Each construct was tested in quadruplicate wells for each of at least 3 independent experiments. Results are normalized to empty vector control (lane 1). Right upper panel: to assess transfection efficiency and cell toxicity, ZAP constructs were cotransfected with CEP-EGFP and fluorescent cells were assayed 4 days later by flow cytometry. Lower panels: Western blot showing expression of tagged ZAP constructs transfected alone. Lower molecular weight bands are considered to be degradation products and are most evident for HA-ZAP-L 253–902 (lane 6). Proteins were sampled at 4 days post-transfection. (C) Expression of HA-ZAP-L inhibits retrotransposition in a dose-dependent manner. Increasing amounts of HA-ZAP-L expressing plasmid, mixed with empty vector to maintain equal DNA concentrations, were cotransfected with 99-PUR-RPS-EGFP in 293T cells and assayed for retrotransposition at 5 days. Results are normalized to empty vector control. Western blot analysis of cytoplasmic lysates (below) shows that even small amounts of exogenous ZAP-L protein can inhibit retrotransposition. Lane 2 shows the background level of endogenous ZAP expression in untransfected 293T cells. (D) Loss of endogenous ZAP expression increases retrotransposition. 293T cells were mock transfected (lane 5) or transfected with scrambled siRNAs (lanes 1 and 2), or sequences directed against ZAP (lanes 3 and 4), and tested for retrotransposition competency of 99-PUR-RPS-EGFP. Bottom panels: Western blots showing that siZC3HAV1-1(1435–1453) and siZC3HAV1-2(1637–1655) decreased endogenous ZAP-L and ZAP-S protein levels by about 90 percent in 293T cells, but had no effect on levels of HSP90. (E) Overexpression of HA-ZAP-L, HA-ZAP-S, rat HA-NZAP, and V5-TEV-MOV10 constructs strongly decreased Alu retrotransposition in HeLa-HA cells. As described in [61], a Ya5 Alu is cloned in a plasmid containing the 7SL pol III enhancer and *neo* ^Tet^ reporter cassette consisting of an antisense copy of a neomycin phosphotransferase gene (*neo*) disrupted by a self-splicing Group I intron. Upon transcription, the intron is spliced out. When this construct is co-expressed with L1 ORF2 (lanes 2 to 7) but not an empty vector (lane 1), Alu RNAs are reverse-transcribed along with the *neo* gene and integrated into the genome to confer neomycin resistance. Following 15 days of treatment with neomycin, resistant colonies were stained and counted. Colony counts are not normalized. (F) Expression of HA-ZAP-L and V5-MOV10 strongly inhibited (lanes 3 and 5), but HA-ZAP-S and HA-ZAP 1–256 (lanes 2 and 4) modestly inhibited mouse IAP element retrotransposition in HeLa-JVM cell culture [62]. Cells were treated with neomycin to select for retrotransposition events. Colony counts are not normalized. On the right are representative T_75_ flasks with Giemsa-stained IAP retrotransposition-positive colonies in the absence (left) or presence (middle and right) of HA-ZAP.

ZAP (PARP13) is a member of the poly(ADP-ribose) polymerase (PARP) family of 17 proteins, some of which are capable of poly(ADP) ribosylation (pADPr) of acceptor proteins using NAD+ as a substrate. Human ZAP is a predominantly cytoplasmic protein that exists in two alternatively spliced isoforms. Long isoform 1 (ZAP-L) possesses a defective C-terminal PARP-like domain that lacks a catalytic glutamate residue essential for pADPr (Fig 2A) [47,48]. This domain is absent in ZAP-S. It was previously demonstrated that the first 254 amino acids of rat ZAP (NZAP), comprising only the four CCCH-type zinc fingers, were sufficient to induce loss of viral mRNA and severely inhibit infection [49]. The zinc finger domain is believed to mediate binding of ZAP to long ZAP-responsive element (ZRE) sequences within the RNAs of target viruses [29,50–52]. Hayakawa et al. [53] reported that ZAP-S, but not ZAP-L, is an ISG whose endogenous expression is increased by Type I interferon, a fact we confirm for 293T cells (Fig 1A, bottom panel).

We cotransfected 99-PUR-RPS-EGFP in 293T cells, together with epitope-tagged ZAP or empty vector, and assayed for fluorescent cells at 5 days post-transfection (Fig 2A and 2B). Coexpressed human HA-tagged HA-ZAP-S and HA-ZAP-L, and SBP-PARP13.1 (ZAP-L with an N-terminal streptavidin binding protein tag [54]) caused a precipitous decrease in the number of retrotransposition-positive 293T cells relative to the empty vector control. L1 inhibition caused by HA-ZAP-S was greater than by V5-TEV-ZAP-S (Figs 1B and 2B), and it is possible that the long tag of the latter construct partially reduced ZAP-S activity. In turn, decrease in retrotransposition was greater for HA-ZAP-L and SBP-PARP13.1 than HA-ZAP-S. ZAP-L inhibited retrotransposition in a dose-dependent manner, and even the lowest levels of ectopically expressed protein caused significant loss of L1 activity (Fig 2C, lanes 3–6). Our data are in line with previous observations that ZAP-L exerts stronger activity against Semliki Forest virus, Sindbis virus, and Moloney leukemia virus (MoLV) than ZAP-S [32,55]. This increased antiviral activity of ZAP-L has been attributed to S-farnesylation within the C-terminal PARP-like domain [56]. Overexpression of PARP1, a nuclear protein, failed to inhibit retrotransposition (Fig 2B, lane 7).

We next tested for the effect of ZAP's N-terminal zinc finger and predicted RNA-binding domain. Expressing only the first 256 residues of human ZAP (HA-ZAP 1–256) reduced L1 retrotransposition to 13 percent of vector control (Fig 2B, lane 5). The analogous N-terminal domain of rat ZAP (HA-NZAP) reduced cell culture retrotransposition even more effectively (to 2.5 percent, although, it should be noted that these truncated ZAP proteins were expressed at higher levels than ZAP-S or ZAP-L; Fig 2B, lower panel).

ZAP constructions with deletions of the zinc finger domain (constructs HA-ZAP-L 253–902 and SBP-PARP13.1^ΔZnF^, which lacks residues 77 to 223) or mutated for five residues considered important for RNA binding (SBP-PARP13.1^VYFHR^) inhibited retrotransposition to a lesser but still significant degree (40–65 percent of vector control; Fig 2B, lanes 6, 9 and 10). Similar results were seen when the retrotransposition assay was performed in HeLa cells (S2A Fig). Together, our data show that the ZAP zinc finger domain alone is sufficient but not exclusive for L1 inhibition.

Tagged ZAP constructs had minimal effect on the expression of cotransfected CEP-EGFP plasmid (Fig 2B, right bar chart). As an additional test for cell toxicity, ZAP constructs or empty vector were cotransfected with a plasmid that constitutively expresses the blasticidin S-resistance gene (*bsr*). Coexpression of HA-ZAP-S, HA-ZAP-L, or rat HA-NZAP did not reduce the number of *bsr*-expressing foci remaining after 10 days of selection with blasticidin (S2B Fig).

We next cotransfected ZAP with ORFeus-HS, a synthetic human LINE-1 construct tagged with the EGFP reporter cassette and containing codon-modified ORF1 and ORF2 sequences and a CMV promoter in place of the L1 5' UTR [57]. Even though ORFeus-HS contains little authentic LINE1 DNA sequence, its retrotransposition in 293T cells was decreased by coexpression of ZAP full-length or N-terminal truncated proteins in the same manner as human L1-RP (S2C Fig). We also assayed ORFeus-Mm [58], a modified version of L1_spa_, a mouse L1 element with low retrotransposition activity [59], possessing both CMV promoter and mouse L1 5' UTR, and codon-optimized to boost its activity to a level similar to that of human L1-RP. ZAP also inhibited cell culture retrotransposition of ORFeus-Mm (S2D Fig). Thus, ZAP suppression of L1s is neither DNA sequence-, promoter-, nor species-specific. We also determined by HIRT preparation plasmid recovery and subsequent PCR that expression of ZAP had no effect on the stability of 99-PUR-RPS-EGFP reporter plasmid DNA (S2E Fig).

We next asked whether endogenous ZAP inhibits cell culture L1 retrotransposition. We confirmed by Western blotting that two previously described siRNA sequences [60] efficiently depleted endogenous ZAP protein when transiently transfected in 293T cells (Fig 2D). Depletion of ZAP enhanced L1 retrotransposition 2- to 3-fold compared with control siRNAs or mock-transfected cells.

We also determined the effect of ZAP expression on retrotransposition of the non-autonomous Alu SINE retrotransposon. Using the assay of Dewannieux et al. [61], we cotransfected in HeLA-HA cells an Alu reporter construct tagged with the neomycin phosphotransferase gene (Alu-*neo* ^Tet^), an ORF2 construct (pCEP-5′UTR-ORF2-No-Neo) to drive retrotransposition, and ZAP constructs or empty vector. Expression of HA-ZAP-L, HA-ZAP-S, rat HA-NZAP, and MOV10 reduced the number of neomycin-resistant retrotransposition-positive colonies more than 80 percent compared with empty vector control. pCEP-5′UTR-ORF2-No-Neo consists of CMV promoter, the L1 5' UTR, and ORF2 sequences only, suggesting that L1 ORF1 or 3' UTR sequence is not essential for ZAP inhibition of retrotransposition.

All human endogenous retroviruses are thought to be incapable of retrotransposition due to inactivating mutations. However, mouse intracisternal A particle (IAP) LTR retrotransposons actively retrotranspose and cause new mutations. Using an established cell culture assay [62], we found that overexpression of HA-ZAP-L and V5-TEV-MOV10 strongly restricted insertion of *neo*-tagged IAP elements in HeLa-JVM cells (Fig 2F). Inhibition by HA-ZAP-S and HA-ZAP 1–256 was less severe, consistent with our results for L1 retrotransposition.

Thus, expression of ZAP inhibits not only retroviruses but also both LTR and non-LTR retrotransposons, including mobile DNA currently active in the human genome.

### ZAP and the L1 RNP associate and colocalize in cytoplasmic granules

We examined the subcellular distribution of ZAP and its association with the L1 RNP. When expressed from a full-length L1 construct, ORF1p is present as an RNP in cytoplasmic stress granules (SGs) together with L1 RNA, ORF2p, and many other RNA-binding proteins. Granules are also detected in cells that express endogenous ORF1p at high levels, and their formation is not dependent upon external stress applied to the cell [63–65]. Stress granules (SGs) are cytoplasmic aggregates that contain stalled 48S pre-initiation complexes and are induced by a range of stresses. P-bodies (PBs) are constitutively expressed cytoplasmic granules rich in factors of RNA decay, including those of RISC [66]. ORF1p granules generally do not overlap, but may juxtapose PBs [63]. Interestingly, we reported [64], and others have confirmed [67], that ORF2p is detected in only a minor subset of ORF1p-positive cells when the two proteins are coexpressed from an L1 construct. The reason for this is unknown.

**Fig 3:**
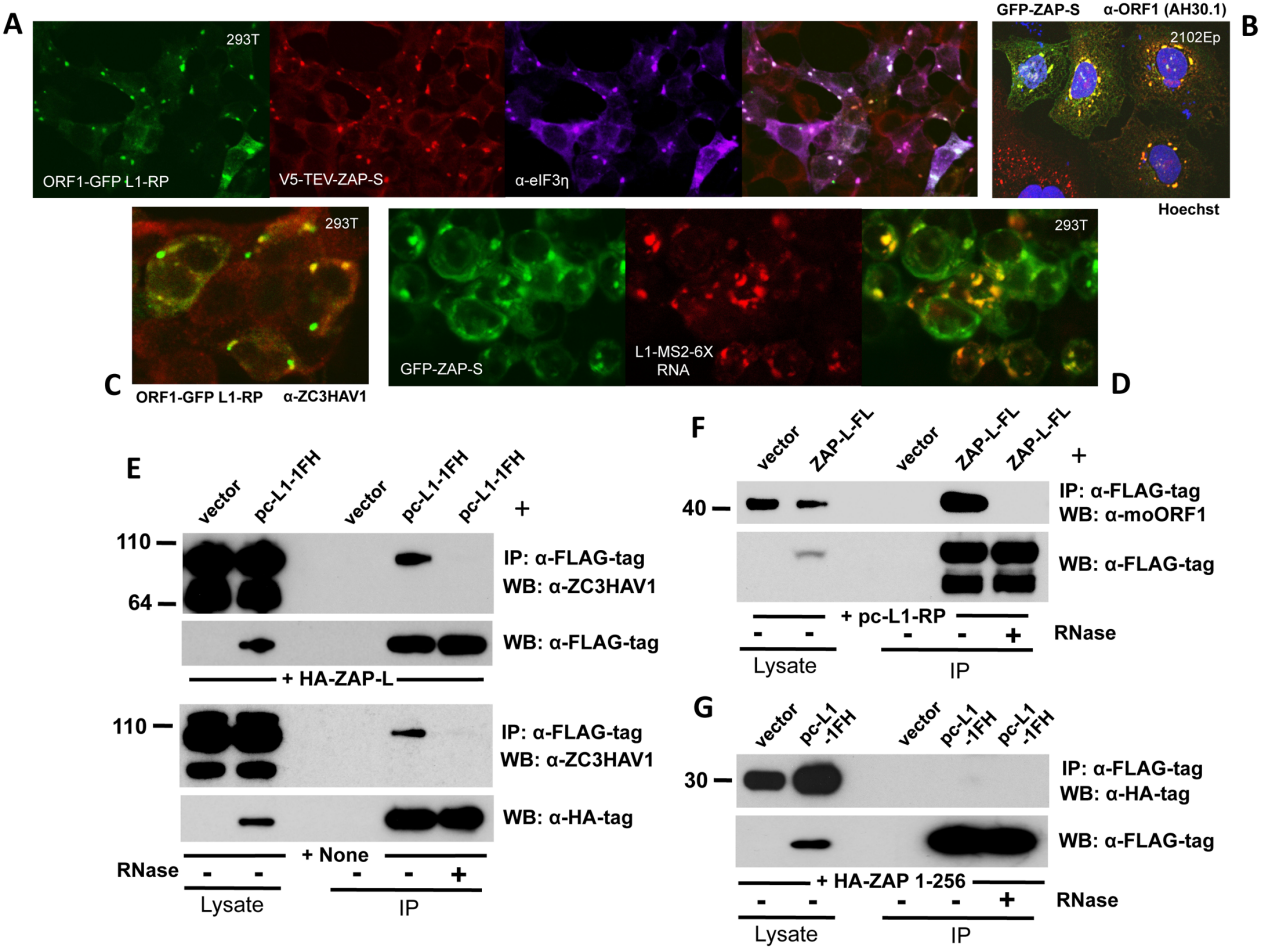
ZAP colocalizes with L1 ORF1 protein in cytoplasmic granules and interacts with its RNP in an RNA-dependent manner. (A) EGFP-tagged ORF1p expressed from the construct ORF1-GFP L1-RP colocalizes with V5- tagged ZAP-S in the cytoplasm and SGs of 293T cells. DyLight 650-conjugated secondary antibody was used to detect endogenous eIF3η, a marker of SGs. (B) GFP-tagged ZAP-S (construct GFP-PARP13.2) colocalizes with endogenous ORF1p in cytoplasmic granules of 2102Ep cells. Cell nuclei were stained with Hoechst 33342. (C) EGFP-tagged ORF1p colocalizes with endogenous ZAP in the cytoplasm of 293T cells. (D) L1 RNA tagged with MS2-repeats and expressed from construct 99-PUR-L1-RP MS2-6X colocalizes with GFP-tagged ZAP-S when detected by antisense RNA-FISH probe Cy3-MS2. (E) Exogenously expressed HA-ZAP-L (top panels) and endogenous ZAP (bottom panels) associate with pc-L1-1FH immunoprecipitates from 293T cells. The associations are lost with RNase treatment. (F) FLAG-tagged ZAP-L (ZAP-L-FL) co-IPs untagged ORF1p expressed from construct pc-L1-RP in 293T cells. (G) Human HA-ZAP 1–256, comprising the N-terminal zinc-finger domain only, fails to co-IP with pc-L1-1FH-encoded ORF1p.

Both ZAP-L and ZAP-S have been reported to colocalize in the cytoplasm with markers of PBs and SGs [52,54,68,69]. Epitope-tagged ZAP-S, coexpressed with ORF1-GFP-L1-RP (a construct with CMV promoter, ORF1 C-terminally tagged with EGFP and intact downstream L1 sequence [64]) and detected by immunofluoresence (IF) of fixed 293T cells, strongly colocalizes with ORF1-GFP and SG marker proteins eIF3 (Fig 3A) and TIA-1. Endogenous ZAP and ORF1p similarly colocalize in the cytoplasm of 293T and 2102Ep cells (Fig 3B and 3C).

To track L1 RNA in fixed cells we used construct 99-PUR-L1-RP-MS2-6X [64], consisting of L1-RP tagged at its C-terminus with a tandem array of six 19-bp stem loop sequences that bind bacteriophage MS2 coat protein. This construct was cotransfected with GFP-tagged ZAP-S and its RNA was detected in fixed cells with a fluorescent *in situ* hybridization (FISH) probe to the MS2 repeats. ZAP-S colocalized with L1 RNA in cytoplasmic granules (Fig 3D).

As noted above, V5-TEV-tagged ZAP-S interacts with L1 RNPs in an RNA-dependent manner (Fig 1D). HA-ZAP-L and endogenous full-length ZAP also co-IP with L1 RNPs expressed from pc-L1-1FH (Fig 3E, upper and lower panels, respectively). Shorter ZAP products, presumed to be degradants, were not recovered in the immunoprecipates. Conversely, FLAG-tagged ZAP-L (ZAP-L-FL) coimmunoprecipitates untagged ORF1p expressed from a full-length L1 construct (Fig 3F). Unexpectedly, despite the ability of the zinc-finger domain alone to strongly inhibit retrotransposons (Fig 2B), we failed to co-IP human HA-ZAP 1–256 (Fig 3G) or rat HA-NZAP with pc-L1-1FH RNPs. Chen et al. [52] proposed that some residues in the zinc finger domain are involved in ZAP's interaction with protein factors rather than RNA. Perhaps an unknown non-L1 protein or RNA can recruit truncated ZAP to the L1 RNP, although this requires further investigation.

In summary, L1 RNPs and ZAP associate and are directed to the same cytoplasmic compartments.

### The ZAP protein interactome

**Fig 4:**
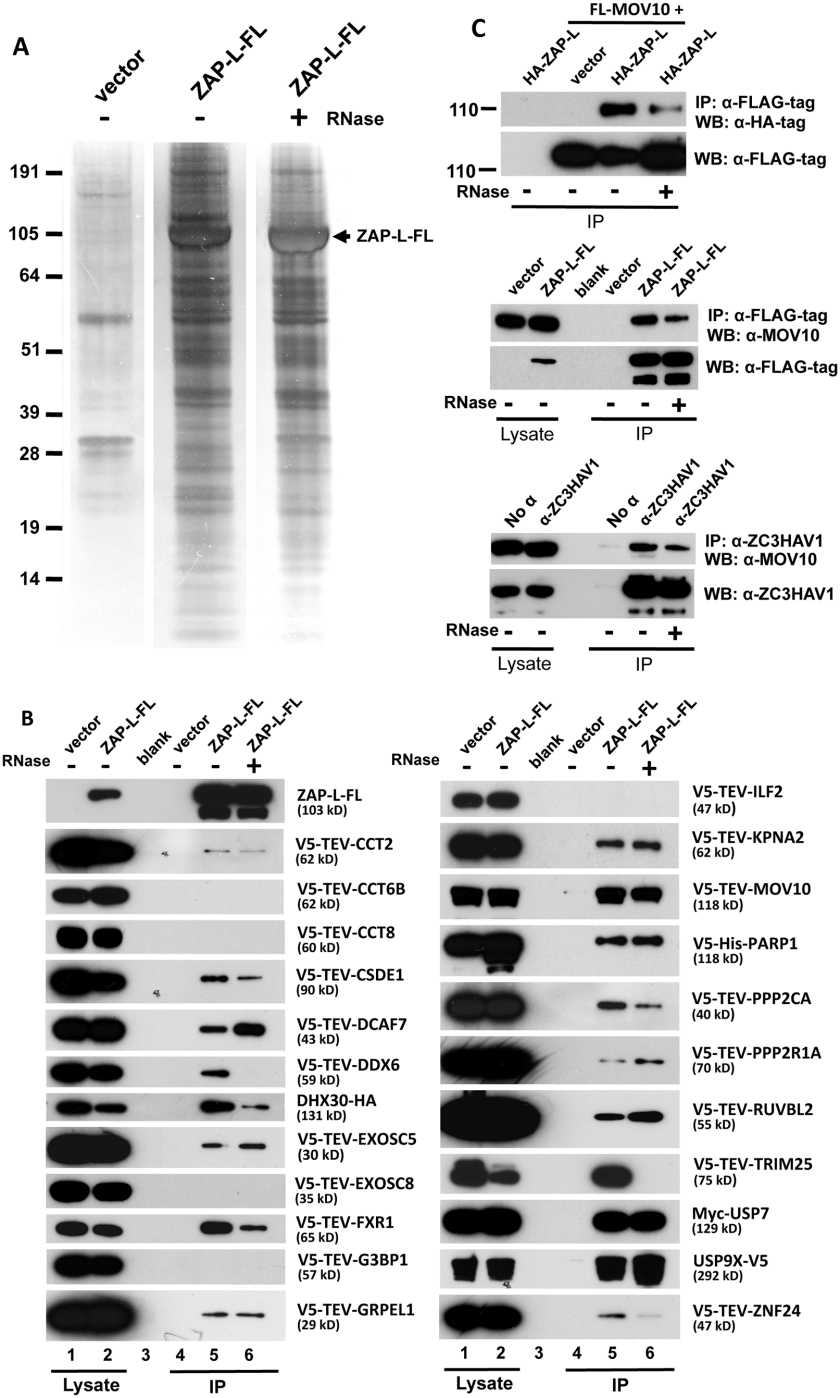
Detection of proteins in the ZAP RNP interactome. (A) ZAP-L-FL or empty vector was immunoprecipitated with α-FLAG agarose from 293T whole cell lysates and resolved on silver-stained polyacrylamide gels. IP reactions were in the presence or absence of RNase. (B) Selected proteins detected in the ZAP interactome were tagged and many were found to specifically co-IP from 293T cells with ZAP-L-FL but not empty vector. Approximately 1% of the input lysate (lanes 1, 2) and 30% of the immunoprecipitate (lanes 4–6) were loaded in gels. All proteins tested for interaction with ZAP-L-FL are shown. IP reactions were in the presence or absence of 50 μg/ml RNase. All test proteins were detected by α-V5 antibody, except USP7 and DHX30, which were detected by α-Myc and α-HA antibodies, respectively. The top-most left panel is representative of tagged ZAP-L-FL present in the input and IP fractions (detected by α-FLAG antibody) and confirms that RNase treatment did not affect ZAP immunoprecipitation on α-FLAG agarose. (Similar results for all of the IP reactions are shown in S4 Fig). Indicated protein molecular weights include the epitope tag. (C) Both endogenous and exogenously expressed ZAP and MOV10 co-IP even in the presence of RNase. Top panels: HA-ZAP-L co-IPs with FLAG-tagged MOV10 on α-FLAG agarose. Middle panel: co-IP of endogenous MOV10 from 293T cells by ZAP-L-FL on α-FLAG agarose. Bottom: Protein G affinity matrix pull-down from 2012Ep cells of endogenous MOV10 and ZAP proteins by α-ZC3HAV1 antibody. The α-ZC3HAV1 antibody (ProteinTech) recognizes the first 351 amino acids of ZAP.

We previously identified 96 proteins associated with the L1 ORF1p RNP and confirmed a subset of these by direct co-IP and subcellular colocalization experiments [46]. We wished to determine the ZAP protein interactome and identify its members that are shared with that of the L1. We transfected ZAP-L-FL and empty vector in parallel in 293T cells and performed IP from whole cell lysates in the presence or absence of RNase. Purification was highly efficient with relatively few proteins identified in vector-only lanes (Fig 4A). Following IP, complex samples were analyzed by tandem mass spectrometry (MS). Excluding 36 ribosomal proteins and likely contaminants (such as keratins), 78 proteins satisfied three criteria: 1) predicted by 3 or more peptides, 2) present in two independent replicate IPs, and 3) unique to ZAP-L-FL isolates. Eleven proteins were shared with the L1 ORF1p RNP interactome defined in our previous paper (Table 1, S1 Table).

**Table 1:** Summary of mass spectrometry analysis of proteins detected with ZAP-L-FL RNP complexes. *

	Category	Protein Name	Gene Symbol	Accession Number	#Unique Peptides	
					Rnase	
					-	+
1		Zinc finger CCCH-type, antiviral 1	ZC3HAV1	NM_020119	72	84
2	Post-translational	Ubiquitin specific peptidase 9, X-linked	USP9X	NM_001039590	24	27
3	modifiers	Protein phosphatase 2, regulatory subunit A, alpha	PPP2R1A	NM_014225	13	16
4		Protein phosphatase 2, regulatory subunit A, beta	PPP2R1B	NM_001177562	9	10
5		Protein phosphatase 2, catalytic subunit, alpha isozyme	PPP2CA	NM_002715	6	7
6		Tripartite motif containing 25/E3 ubiquitin/ISG15 ligase TRIM25	TRIM25	NM_005082	6	2
7		Ubiquitin specific peptidase 7 (herpes virus-associated)	USP7	NM_003470	2	3
8		Poly [ADP-ribose] polymerase 1	PARP1	NM_001618	0	3
9	Chaperonin	Chaperonin containing TCP1, subunit 2 (beta)	CCT2	NM_001198842	17	16
10		Chaperonin containing TCP1, subunit 6A (zeta 1)	***CCT6A***	NM_001762	10	6
11		T-complex protein 1, alpha subunit	CCT1	NM_030752	9	6
12		Chaperonin containing TCP1, subunit 3 (gamma)	CCT3	NM_005998	9	6
13		Chaperonin containing TCP1, subunit 4 (delta)	***CCT4***	NM_006430	7	7
14		Chaperonin containing TCP1, subunit 7 (eta)	CCT7	NM_006429	6	8
15		DnaJ (Hsp40) homolog, subfamily A, member 1	DNAJA1	NM_001539	5	7
16		Heat shock 105kDa/110kDa protein 1	HSPH1	NM_006644	2	4
17		Heat shock 70kDa protein 4	HSPA4	NM_002154	1	7
18		DnaJ (Hsp40) homolog, subfamily A, member 2	DNAJA2	NM_005880	1	4
19	RNA Helicase	DEAH (Asp-Glu-Ala-His) box polypeptide 30	DHX30	NM_014966	23	6
20		DEAD (Asp-Glu-Ala-Asp) box polypeptide 6	DDX6	NM_004397	5	0
21		DEAH (Asp-Glu-Ala-Asp/His) box polypeptide 57	DHX57	NM_198963	4	0
22		DEAD (Asp-Glu-Ala-Asp) box polypeptide 18	DDX18	NM_006773	3	0
23		Moloney leukemia virus 10, homolog (mouse)	***MOV10***	NM_020963	3	1
24		YTH domain containing 2	YTHDC2	NM_022828	3	0
25	SG/PB proteins	Fragile X mental retardation, autosomal homolog 1	FXR1	NM_005087	7	7
26		ELAV (embryonic lethal, abnormal vision, Drosophila)-like 1 (Hu antigen R)	***ELAVL1***	NM_001419	3	1
27		GTPase activating protein (SH3 domain) binding protein 1	G3BP1	NM_198395	3	0
28		GTPase activating protein (SH3 domain) binding protein 2	G3BP2	NM_203504	3	0
29		Fragile X mental retardation, autosomal homolog 2	FXR2	NM_004860	1	3
30	mRNA decay	5'-3' exoribonuclease 2	XRN2	NM_012255	12	6
31		Exosome component 8	EXOSC8	NM_181503	3	2
32		YTH domain family, member 2	YTHDF2	NM_016258	3	2
33	Chromatin	Chromodomain helicase DNA binding protein 4	CHD4	NM_001273	6	7
34	binding/	Histone deacetylase 1	HDAC1	NM_004964	3	2
35	remodelling	Heterochromatin protein 1-binding protein 3	HP1BP3	NM_016287	3	0
36		RuvB-like 1 (E. coli)	RUVBL1	NM_003707	2	3
37		RuvB-like 2 (E. coli)	RUVBL2	NM_006666	2	3
38	Zinc finger	Zinc finger protein 326, transcript variant 1	ZNF326	NM_182976	4	9
39	transcription	Zinc finger RNA binding protein	ZFR	NM_016107	4	0
40	factors	Zinc finger protein 24	ZNF24	NM_006965	2	3
41	Others	Sjogren syndrome antigen B (autoantigen La)	***SSB***	NM_003142	20	0
42		Ribosomal L1 domain containing 1	RSL1D1	NM_015659	19	0
43		Endoplasmic reticulum protein 44	ERP44	NM_015051	15	18
44		Carbamoyl-phosphate synthetase 2, aspartate transcarbamylase	CAD	NM_004341	11	31
45		Interleukin enhancer-binding factor 2	***ILF2***	NM_004515	11	7
46		Pentatricopeptide repeat domain 3	PTCD3	NM_017952	11	11
47		GrpE-like 1, mitochondrial (E. coli)	GRPEL1	NM_025196	10	11
48		Guanine nucleotide binding protein-like 3 (nucleolar) /nucleostemin	GNL3	NM_014366	9	2
49		GTP binding protein 4	GTPBP4	NM_012341	9	0
50		General transcription factor IIi	GTF2I	NM_032999	8	15
51		Insulin-like growth factor 2 mRNA binding protein 3	IGF2BP3	NM_006547	8	0
52		Glutamyl-prolyl-tRNA synthetase	EPRS	NM_004446	7	9
53		KRI1 homolog (S. cerevisiae)	***KRI1***	NM_023008	7	0
54		Heterogeneous nuclear ribonucleoprotein U-like 1	HNRPUL1	NM_007040	6	0
55		Ly1 antibody reactive	LYAR	NM_017816	6	0
56		NOP2 nucleolar protein	NOP2	NM_001258308	6	0
57		Ribonuclease/angiogenin inhibitor 1	RNH1	NM_002939	6	0
58		Apoptosis-inducing factor, mitochondrion-associated, 1	AIFM1	NM_001130846	5	4
59		La ribonucleoprotein domain family, member 1	***LARP1***	NM_015315	5	0
60		Reticulocalbin 2, EF-hand calcium binding domain	RCN2	NM_002902	5	4
61		Cell cycle associated protein 1	CAPRIN1	NM_005898	4	0
62		FtsJ homolog 3 (E. coli)	FTSJ3	NM_017647	4	0
63		Heterogeneous nuclear ribonucleoprotein A0	HNRNPA0	NM_006805	4	0
64		La ribonucleoprotein domain family, member 7	LARP7	NM_015454	4	0
65		MYB binding protein (P160) 1a	MYBBP1A	NM_014520	4	3
66		Ribosomal RNA processing 1B	RRP1B	NM_015056	4	0
67		Coiled-coil domain containing 86	CCDC86	NM_024098	3	0
68		Cold shock domain containing E1, RNA-binding	CSDE1	NM_001130523	3	0
69		Karyopherin alpha 2 (RAG cohort 1, importin alpha 1)	***KPNA2***	NM_002266	3	3
70		Staufen double-stranded RNA binding protein 1	***STAU1***	NM_004602	3	0
71		Topoisomerase (DNA) I	***TOP1***	NM_003286	3	0
72		A kinase (PRKA) anchor protein 8	AKAP8	NM_014371	2	3
73		Tu translation elongation factor, mitochondrial	TUFM	NM_003321	0	6
74		DDB1- and CUL4-associated factor 7	DCAF7	NM_005828	0	3
75		General transcription factor IIIC, polypeptide 1, alpha 220kDa	GTF3C1	NM_001520	0	3
76		General transcription factor IIIC, polypeptide 3, 102kDa	GTF3C3	NM_012086	0	3
77		Pyrroline-5-carboxylate reductase-like	PYCRL	NM_023078	0	3
78		RNA binding motif protein 14	RBM14	NM_006328	0	3

* Only non-ribosomal proteins predicted by three or more unique peptides, present in two independent co-IP reactions, and not detected in empty vector control isolates are shown. Proteins are organized within selected functional categories and ordered within each category by number of unique peptides identified in the IP reaction not treated with RNase. Detailed MS data are presented in S1 Table.

Gene Symbol: Proteins shared with the L1 ORF1p interactome [46].

To confirm protein interactions, a subset of cDNAs of proteins identified by MS were subcloned with an N-terminal V5-TEV-epitope tag or were obtained as gifts. Following cotransfection in 293T cells, 18 of 23 proteins tested co-IPed with ZAP-L-FL on α-FLAG agarose (Fig 4B). A majority of these interactions were resistant to RNase digestion, suggesting direct protein-protein binding. In contrast, our previous work characterizing the ORF1p interactome found almost all of 41 confirmed protein associations to be lost upon RNase treatment [46].

Our analyses identified most previously described ZAP-interacting proteins. We confirmed RNA-independent binding of ZAP and PPR2R1A (PR65A) [70], a component of the protein phosphatase 2 (PP2A) complex, and show for the first time RNase-resistant binding of its catalytic subunit, PPP2CA, as well (Fig 4B). Wang et al. [70] reported that knockdown of PPR2R1A by shRNAs reduced ZAP suppression of an MoLV reporter. It has been reported that several serine residues immediately downstream of the rat ZAP zinc-finger domain are phosphorylated by glycogen synthase kinase 3β (GSK3β), and that overexpression of GSK3β reduces, and its inhibition increases ZAP activity against MoLV [71]. These data are all consistent with inhibition of ZAP antiviral function by phosphorylation. Our MS analyses of human ZAP protein sequence (65% coverage) confirmed two phosphorylated residues (S257 and S284) homologous to those detected by Sun et al. [71] in rats, and identified four additional phosphorylated sites (S335, T375, S378, S796). However, a role for phosphorylation or PP2A-mediated dephosphorylation in modulating ZAP inhibition of retrotransposition remains to be determined.

**Fig 5:**
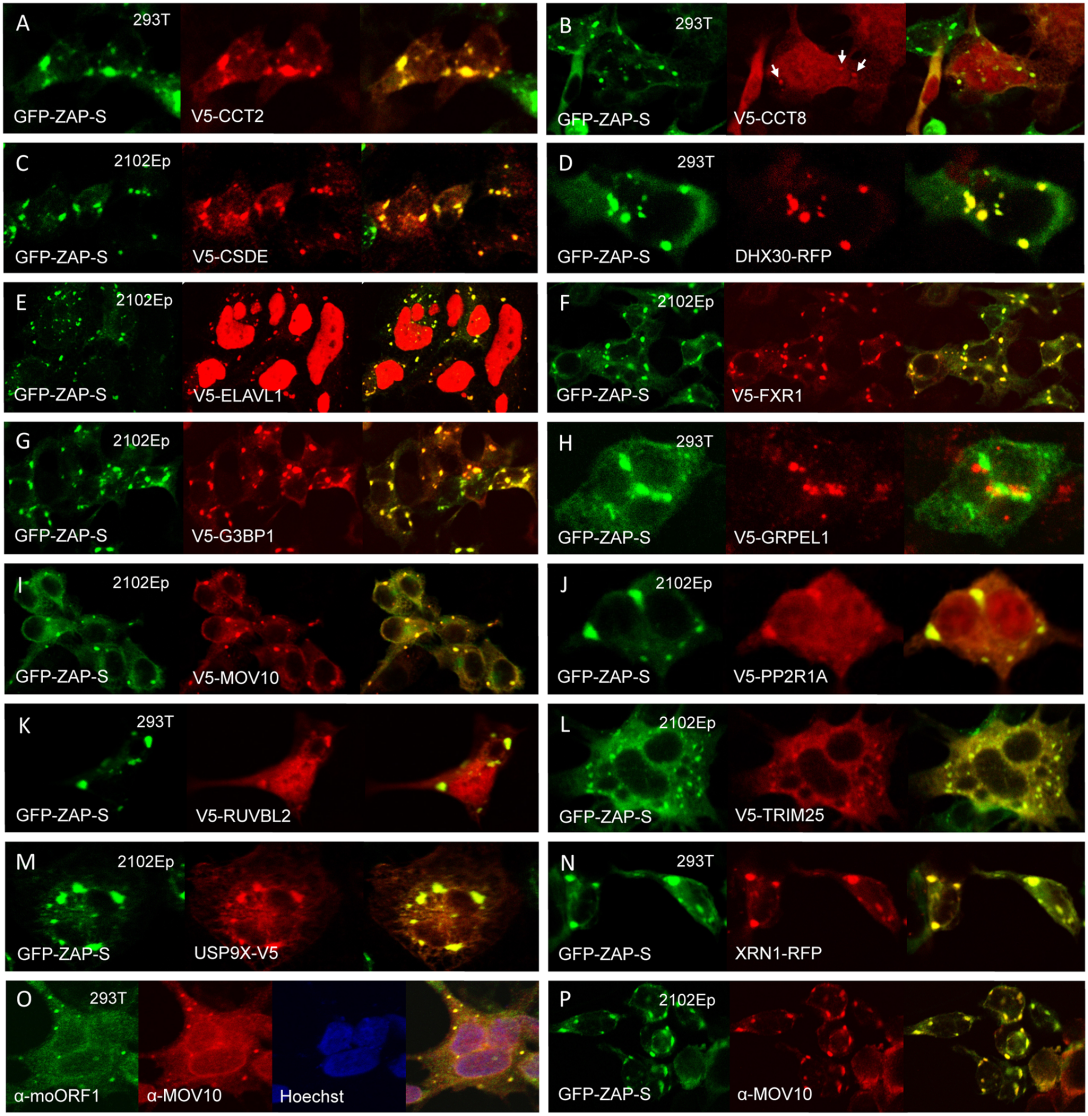
Many members of the ZAP interactome colocalize with GFP-tagged ZAP-S in cytoplasmic granules of unstressed 293T cells. (A–N) Construct GFP-PARP13.2 was co-transfected with V5-tagged proteins in all cases except (D) DHX30 and (N) XRN1, which were tagged with red fluorescent protein (RFP). The cell type is indicated in each panel. (O) Endogenous ORF1p and MOV10 co-localize in cytoplasmic granules. (P) Endogenous MOV10 protein co-localizes with GFP-ZAP-S.

It has been proposed that ZAP recruits the 3'-5' exosome to degrade target viral RNAs in cytoplasmic granules [51,72]. We identified exosome component EXOSC8 (RRP43) in ZAP RNP immunoprecipitates, although we could not confirm its direct binding with ZAP-L-FL (consistent with [72]). According to previous reports, EXOSC5 (RRP46) binds rat but not human ZAP [50,72]. However, when tested, we found EXOSC5 to bind weakly with human ZAP-L-FL in the presence or absence of RNase. We also identified as a strong ZAP interactor DHX30, an RNA helicase believed to be recruited by ZAP to unwind viral RNAs and facilitate their exosome-mediated degradation in stress granules [69,73]. We confirmed colocalization of DHX30 with both ZAP-S and ORF1-GFP in cytoplasmic granules, and RNA-independent binding of DHX30 with ZAP-L-FL (Figs 4B and 5D and S3A).

In addition to the 3'-5' degradation exosome, 5′-3′ degradation enzymes, including 5'-3' Exoribonuclease 1 (XRN1) and Poly(A)-Specific Ribonuclease (PARN), have been reported to bind ZAP and augment antiviral function [51]. We detected the XRN1 paralog XRN2 in the ZAP interactome. XRN2 not being available, we tested XRN1 tagged with RFP and found it to colocalize with GFP-tagged ZAP-S in cytoplasmic granules (Fig 5N). XRN1 is a known SG and PB component and also colocalizes with ORF1p-GFP in SGs [63,74].

We also discovered novel ZAP-associated proteins. In addition to PP2A complex members, ZAP RNPs contain several other proteins involved in post-translational modification (PTM). TRIM25, which functions as an E3 ubiquitin and ISG15 ligase and defends against viruses by mediating ubiquitination of RIG-1/DDX58 [75], associates with ZAP in the presence of RNA (Fig 4B). Even after RNase digestion, ZAP-L strongly binds two ubiquitin carboxyl-terminal peptidases, USP7 and USP9X. Our MS analyses predicted two ubiquitinated lysines (K226, K783) and the UbPred and CKSAA_UBSITE algorithms [76,77] predicted 6 additional sites of ubiquitination within ZAP-L. Perhaps ubiquitin peptidases associate with the ZAP RNP to enhance its anti-retroelement activity by limiting ubiquitin-mediated degradation, although that remains to be determined.

While its own PARP domain is likely catalytically inactive, ZAP-L itself is ADP-ribosylated and known to recruit other PARPs that are capable of pADPr, such as PARP5 and PARP12 [68]. PARP1 is the founding member of the PARP family. We report for the first time the direct binding of ZAP and PARP1 independent of RNase digestion. It is not known if PARP1 ribosylates ZAP.

Also identified within the ZAP RNP were almost all components of the cytosolic chaperonin-containing TCP1 (CCT) complex. We tested several CCT complex members and confirmed weak binding of CCT2, but not CCT6B or CCT8, with ZAP-L-FL (Fig 4B). The CCT complex was first discovered for its critical role in the folding of actin and tubulin, and subsequently many other CCT binding proteins were reported [78,79]. This is the first report of the association of CCT and ZAP. CCT4 and CCT6 were also detected in the ORF1p interactome [46].

The ZAP interactome includes several canonical components of SGs (FXR1, FXR2, G3BP1, and ELAVL1) and PBs (DDX6). We confirmed the association of these proteins with ZAP by colocalization in cytoplasmic granules and/or co-IP (Figs 4B and 5). Indeed, 65 percent (12/18) of the proteins tested that directly immunoprecipitated with ZAP-L-FL (Fig 4B) also colocalized with GFP-tagged ZAP-S in cytoplasmic granules of 293T cells (Fig 5). To the best of our knowledge, CCT2, CCT8, PPP2R1A, TRIM25, and USP9X have not previously been reported in granules (Figs 5A, 5B, 5J, 5L and 5M).

We screened test proteins for colocalization in cytoplasmic granules with ZAP-S rather than ZAP-L (Fig 5), and it is possible that additional ZAP-associated proteins, bound only by ZAP-L's PARP-like domain, escaped detection. While ZAP-L and ZAP-S colocalize in cells, it is of interest that overexpression of ZAP-L induces fewer and larger cytoplasmic aggregates, and apparently binds ZAP-S to cause its redistribution to these large foci when the two isoforms are coexpressed; ZAP is known to dimerize (S3B Fig; [52]). Lee et al. [69] reported that murine ZAP-S colocalizes in the cytoplasm with markers of PBs and SGs, but not with markers of mitochondria, endosomes, peroxisomes, or lysosomes. On the other hand, Charron et al. [56], found that C-terminal S-farnesylation of mouse ZAP-L caused its partial redistribution to lysosomes and late endosomes. Reasons for the differing patterns of ZAP-S and ZAP-L bear further investigation.

We previously reported that endogenous L1 ORF1p and antiviral protein MOV10 associate in an RNA-dependent manner and colocalize in SGs of cells (Fig 5O; [27]). We now show that MOV10 is also a component of the ZAP interactome. Recently, Gregersen et al. [80] also detected ZAP by SILAC (stable isotope labeling by amino acids in cell culture) analyses of MOV10-interacting proteins. Both endogenous and exogenously expressed ZAP and MOV10 co-IP in a manner partially resistant to RNase digestion (Fig 4C). We could not detect binding of MOV10 with HA-ZAP-L 1–256 or rat HA-NZAP. ZAP and MOV10 proteins closely colocalize in cytoplasmic granules (Fig 5I and 5P).

In summary, we present for the first time a comprehensive analysis of the ZAP protein interactome. Most of its components we tested directly bound and/or colocalized in RNA granules with ZAP. ZAP may recruit many cellular proteins to these cytoplasmic structures (or vice versa).

### ZAP restricts L1 RNPs

**Fig 6:**
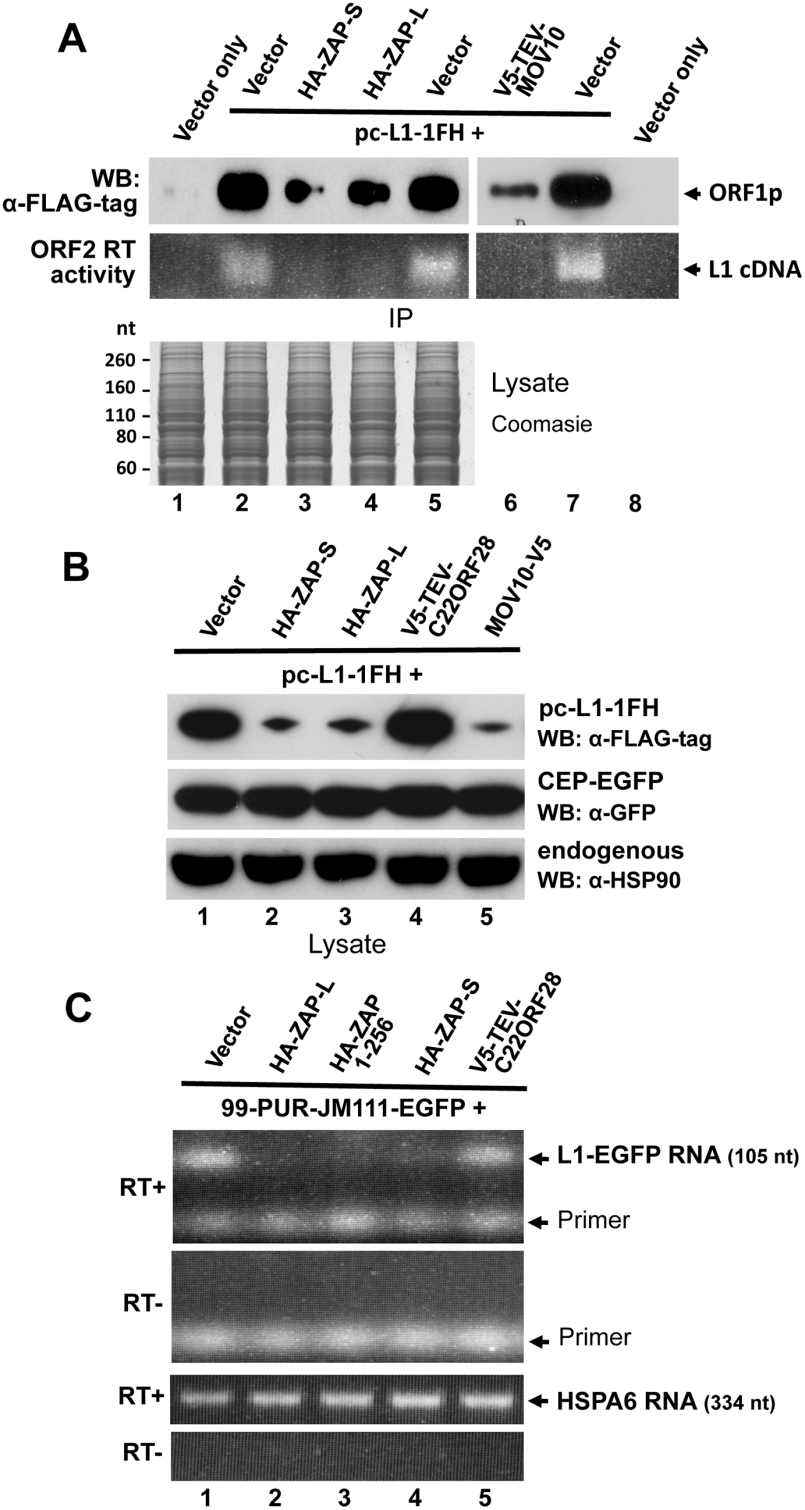
ZAP inhibits exogenous L1 RNP levels in cells. (A) Expression of HA-ZAP-L, HA-ZAP-S, and V5-TEV-MOV10 reduces the amount of ORF1 protein (top IP panel) and ORF2p RT activity (bottom IP panel) in α-FLAG antibody purified pc-L1-1FH immunoprecipitates. RT activity was determined by the LEAP assay [81]. Below: Coomasie-stained gel of cell lysates prior to IP showed no obvious loss of global protein levels in the presence of ZAP. (B) Analysis of 293T cell lysates showing that ORF1 protein from pc-L1-1FH is reduced in the presence of ZAP and MOV10, but not empty vector or an unrelated protein, C22ORF28 (top panel). Panels below show by Western blotting that neither ZAP nor MOV10 alter expression of endogenous HSP90 or GFP protein expressed from cotransfected CEP-EGFP. Lysates were prepared from two pooled wells of a six-well plate. (C) Expression of ZAP causes loss of L1 RNA. Top panels: RT-PCR of L1 RNA expressed from 99-PUR-JM111-EGFP, which expresses L1-RP with an ORF1 mutation that prevents genomic insertion. PCR primers spanned the intron of the EGFP reporter cassette to distinguish spliced RNA products from contaminating plasmid DNA (generating a 105 nt band vs a 1 kb band). PCR reactions are shown in the presence or absence of RT. Bottom panels: Levels of endogenous HSPA6 RNA were the same in the presence or absence of ZAP.

Previously, we demonstrated that overexpression of MOV10 strongly reduces the steady state number of L1-encoded RNA and proteins in transfected cells, although the mechanism of this loss remained uncertain [64]. We similarly assayed the effects of ZAP on L1 expression. As with MOV10, levels of ORF1 protein expressed from pc-L1-1FH were significantly reduced in L1 RNPs immunoprecipitated from cytoplasmic extracts in the presence of cotransfected ZAP-L and ZAP-S. ORF2 activity was almost undetectable in the LEAP assay for L1 reverse transcription (Fig 6A, lanes 3 and 4; [81]. In whole cell lysates, ORF1p expressed from pc-L1-1FH was significantly lower in the presence of ZAP-S, ZAP-L, or MOV10 but not empty vector or an unrelated protein (Fig 6B). This was not a general protein effect, as overexpression of ZAP did not affect levels of coexpressed EGFP or endogenous heat shock protein 90 (Fig 6B), and was without obvious effect on global protein expression detected by Coomassie blue staining of cell lysates (Fig 6A, lower panel).

We next examined L1 RNA transcribed from construct 99-PUR-JM111-EGFP in the presence or absence of ZAP. This construct contains a mutation in L1 ORF1 that prevents genomic insertions. PCR primers flanked the intron of the EGFP reporter cassette allowing products amplified from spliced cDNA to be distinguished from those of contaminating plasmid DNA. Paralleling the loss of ORF1 protein, diminished levels of L1 RNA were detected by RT-PCR in whole cell lysates cotransfected with HA-ZAP-L HA-ZAP-S, or HA-ZAP 1–256, but not with empty vector or an unrelated protein. Analysis of endogenous HSPA6 RNA showed no such effect (Fig 6C). We failed to determine whether or not ZAP affects expression of endogenous L1s in the genome. The observed reduction of exogenous L1 RNA in the presence of ZAP protein is consistent with previous reports that ZAP inhibits infecting viruses by causing loss of their RNAs at a post-transcriptional step [29,49,51,72,82].

## Discussion

Restriction factor proteins are part of the innate immune defense system of the cell, which often detects infection by receptors that recognize viral nucleic acids. Many of these factors target retroviruses, but some, such as ZAP, act against a wide range of viral families. In certain cell types expression of antiviral genes is induced by type I or type II interferons. We have expanded the list of IFN-stimulated genes that limit LINE-1 retrotransposition when overexpressed, including BST2, ISG20, MAVS, MX2, and ZAP (Fig 1B).

Most dramatic was an almost complete loss of retrotransposition in the presence of MAVS protein, which is only partly explained by cytotoxicity. MAVS acts downstream of the RIG-I and IFIH1 cytoplasmic receptors for viral dsRNAs. Receptor activation causes multimerization of MAVS to trigger a signaling cascade and production of type I IFNs [83]. Recent evidence indicates that upon viral detection peroxisomal-localized MAVS rapidly induces interferon-independent expression of defense factors to provide short-term protection. Mitochondrial MAVS signaling occurs later in infection, triggering IFN expression and induction of ISGs, and sustaining the immune response [84]. Thus, the profound inhibition of retrotransposition caused by overexpressed MAVS is likely due to the combined action of a number of interferon-induced genes.

ISG20 strongly inhibited L1 retrotransposition without obvious cytotoxicity caused by its overexpression. ISG20 is a 3'-5' exonuclease that inhibits replication of several human and animal RNA viruses, including HIV-1 [7,85,86]. Its mechanism of action is unknown, although one might assume that it degrades viral or retrotransposon RNA

Inhibition of L1 retrotransposition by the dynamin-like GTPase MX2, but not by its closely related paralog MX1, parallels recent reports that MX1, a broad-spectrum inhibitor of many RNA and DNA viruses including influenza virus, fails to inhibit HIV retrovirus. On the other hand, MX2 does not inhibit infuenza virus but inhibits multiple strains of HIV1 at a late post-entry step by targeting the viral capsid and preventing accumulation of viral cDNA in the nucleus. [41–43,45]. Our mutation analyses suggest that the mechanism of MX2 inhibition of L1 retrotransposition may differ in some aspects from retroviral inhibition, requiring GTP binding but not nuclear localization.

Like MX1, overexpression of TRIM28/KAP1 had little effect on retrotransposition (Fig 1B). However, in mouse ES cells TRIM28 strongly silences expression of multiple classes of endogenous LTR retroelements and modestly suppresses L1s by recruiting chromatin-remodeling factors [87,88]. Thus, TRIM28 may inhibit human retrotransposition by epigenetic modification of endogenous L1 elements, but remain ineffective in our cell culture assay against L1s expressed from a plasmid.

Importantly, we showed that transient expression of either the long or short isoform of a general protein inhibitor of viral infection, ZAP, potently restricts genomic insertion of both non-LTR and LTR retrotransposons. Furthermore, siRNA-mediated knockdown of endogenous ZAP increased L1 retrotransposition 2- to 3-fold in 293T cells. Association with the L1 complex is confirmed by close colocalization of ZAP protein with ORF1p and L1 RNA in cytoplasmic stress granules, and by the detection of ZAP in RNP particles captured by immunoprecipitation of a tagged L1 construct.

The CCCH-type zinc finger domain of ZAP recognizes MoLV and other viral transcripts and induces their degradation by recruiting RNA decay proteins [50,51,69,72,73]. Selected cellular RNAs are also targeted, including the TRAIL receptor, TRAILR4 [54]. Our evidence suggests that RNA degradation is also a characteristic of ZAP-associated loss of retrotransposition. Levels of exogenously expressed L1 RNA and protein are reduced in cell lysates in the presence of ZAP (Fig 6C). Loss of ZAP binding in the L1 RNP upon RNase treatment, and the fact that deletion of the zinc finger domain or mutation of residues considered important for its RNA binding significantly reduced inhibition of retrotransposition, suggests that ZAP binds the L1 RNA to promote loss of RNP integrity. However, we cannot exclude the possibility that ZAP binds some other RNA that itself is recruited to the L1 RNP. Many non-L1 RNA species have been found in association with the L1 ORF1p complex, including mRNAs, Alu, SVA, and small cytoplasmic and nuclear RNAs [46,89]. ZAP binds ZREs with a minimum known length of 500 bp, and no common motif or secondary structure has been found [29,50,51]. A detailed investigation of how ZAP binds L1 RNA is required. The fact that deletion or mutation of the zinc finger RNA-binding domain reduced but did not abolish ZAP inhibition of retrotransposition (Fig 2B), suggests that a second RNA-binding domain may exist, or that protein-protein interactions are also important for retrotransposon inhibition.

Our data cannot exclude the possibility that ZAP may also inhibit L1s at the protein level (perhaps by binding L1 RNA to interfere with ORF translation). Effects of ZAP on viral translation have been described [31,51]. Inhibition of GSK3β phosphorylation increases ZAP's ability to repress target mRNA translation without increased mRNA degradation [71]. And recently, Zhu et al. [82] demonstrated that ZAP represses translation of HIV-1 and Sindbis virus reporter constructs independently of mRNA decay by directly binding translation initiation factor eIF4A and interfering with its interaction with eIF4G.

MOV10 is an RNA helicase that also strongly inhibits retrotransposition in cell culture assays [26,27,90]. Li et al. [28] showed that overexpression of MOV10 strongly reduced levels of exogenously expressed IAP and L1 RNA at a post-transcriptional step, while inhibition of endogenous MOV10 increased L1 RNA. On the other hand, Lu et al. [90] found that MOV10 decreases IAP RT products but not levels of IAP RNA and protein. The facts that MOV10 and ZAP bind each other independently of RNA, colocalize in cytoplasmic granules, associate with the L1 RNP, and promote similar loss of L1 RNP integrity and retrotransposition in cells, suggest that the two proteins may function in a common pathway, a notion worthy of further investigation.

In this study, we also present for the first time a detailed analysis of the ZAP protein interactome, confirming most of its previously known interacting proteins and identifying new member proteins whose association with the ZAP RNP is consistent with its known cellular functions. For example, close colocalization in cytoplasmic granules of many of these proteins with ZAP, including L1 ORF1p, is not surprising in light of ZAP's dual roles in the assembly of RNA granules and the control of microRNA silencing. While itself catalytically inactive, ZAP recruits other PARPs active for poly(ADP)-ribosylation which is critical for SG formation [68,91]. Furthermore, overexpression of ZAP causes loss of microRNA silencing by targeting Ago2 (a binding partner of ZAP, although not one detected by our study) for pADPr [91,92].

We detected several PTM-related proteins within the ZAP RNP, including protein phosphatase 2A complex members (PPP2R1A/B and PPP2CA), two related deubiquitinating enzymes (USP7 and USP9X), and TRIM25 (an E3 ubiquitin/ISG15 ligase). In addition to pADPr [68], Sun et al. [71] reported phosphorylation of ZAP by GSK3β, and Charron et al. [56] found that S-farnesylation at the C-terminus of ZAP-L enhanced its restriction of Sindbis virus. Thus, PTMs likely modulate ZAP antiviral activity and may explain why ZAP efficiently restricts some classes of virus but not others.

We also identified epigenetic modifying enzymes in ZAP-L-FL immunoprecipitates (Table 1). RUVBL1 and RUVBL2 are conserved AAA+ protein ATPases present in histone acetyltransferase complexes NuA4 and Tip60, chromatin remodelling complexes Ino80 and SWR-C, and the telomerase complex [93]. HDAC1 and CHD4 are components of the NuRD (nucleosome remodeling and histone deacetylase) complex, along with HP1, SETDB1 and TRIM28 (KAP1). While we detected TRIM28 in ZAP immunprecipitates, it was also present in vector-only samples (11 vs 4 peptides, respectively) and is excluded from Table 1. TRIM28 is targeted to specific DNA sequences via its interactions with various zinc finger proteins, and mediates gene silencing by recruiting NuRD to target promoters [94,95]. Epigenetic modifications involving TRIM28 and SETDB1 have been implicated in silencing both endogenous retroviruses and retrotransposons, including multiple classes of LTR retroelements and L1s [88,89,96–100]. Reichman et al. [101] also showed *in silico* that HDAC1 silences LTR retrotransposons in mouse mES cells. A possible link between ZAP and epigenetic silencers deserves investigation. Although ZAP is predominantly a cytoplasmic protein it can also function in the nucleus [29,102].

Macdonald et al. [103] showed that an unknown IFN-induced factor(s) synergizes with ZAP to control viral infection. Karki et al [104] identified 16 IFN-stimulated genes that act synergistically with ZAP to reduce alphavirus infectivity, including IFIH1 and SAMHD1. Furthermore, ZAP-S stimulates RIG-1-mediated production of Type I interferon [53]. It is remarkable that 34 of the 78 proteins of the ZAP interactome shown in Table 1 are identified in the Interferome v2.01 Database of about 2000 ISGs as being induced at least 2-fold by interferon in either mice or humans (S1 Table; [105]). This is a four-fold enrichment of ISG proteins in the ZAP interactome over what would be expected by chance. Likely we would have detected an even greater number of ISG products within the ZAP-L-FL interactome if prior to IP we had first stimulated their expression with interferon. MX1 and, MX2, for example, while not detected by our MS analyses of endogenous ZAP-interacting proteins, when overexpressed strongly bind ZAP-L-FL in a manner resistant to RNase treatment (S5 Fig). The association of ZAP with many ISG products suggests it may be a key player in the interferon response. This idea is supported by a recent study showing that ZAP-L-depleted HeLa cells are strongly enriched for expression of genes in the interferon immune response pathway [54].

In addition to MOV10, other IFN-stimulated proteins associated with the ZAP RNP complex also play roles in viral control. Overexpression of DHX30, for example, strongly enhances expression of HIV-1, while restricting its RNA packaging [106]. G3BP1 and G3BP2 regulate expression of ISGs known to have broad antiviral activity, and are required for an IFN response against dengue and yellow fever viruses [107]. Chaperone HSPA4 (HSP70) inhibits viral gene expression and replication [108]. Its co-chaperone DNAJ (HSP40) has been associated with both activation and inhibition of viruses, including HIV [109]. Ubiquitination by TRIM25 increases the ability of RIG-1 to initiate antiviral signaling by facilitating its interaction with MAVS. Influenza A virus nonstructural protein 1 specifically inhibits TRIM25-mediated RIG-I ubiquitination, thereby suppressing its antiviral activity [75,110]. USP7 was originally identified by its interaction with the HSV-1-encoded E3 ubiquitin ligase ICP0 [111]. PARP1 is required for efficient replication and integration of HIV-1 [112,113], and has been implicated in repressing Epstein Bar virus, Kaposi's sarcoma-associated herpesvirus, and *Drosophila* retrotransposons [114–116]. RUVBL2 inhibits influenza virus replication, apparently by interfering with oligomerization of the viral nucleoprotein [117]. Future investigations of the associations of these proteins with the ZAP complex could yield new insights into antiviral restriction.

As we showed in Fig 1A, application of Type I interferon represses retrotransposition in cell culture. While this paper was under review, another study was published showing that increased endogenous L1 expression in the testes of MOV10L-deficient mice, or transfection of L1 constructs in mouse embryonic fibroblasts, is marked by increased levels of INFβ [118]. MOV10L is a restriction factor that represses retroelements in the male germline [119]. Conversely, treatment of human cells with INFβ suppressed L1 replication. Functional loss of an anti-retroelement restriction factor can alter the normal metabolism of retrotransposon RNA or its reverse-transcribed cDNA with possible consequences for the organism. Such may be the case with Type I interferonopathies, which include AGS, SLE, spondyloenchondrodysplasia, and STING-associated vasculopathy [120 121]. Indeed, it has been proposed that misregulated nucleic acids, deriving from an as yet unknown endogenous source, but possibly retrotransposons, accumulate and are recognized by sensors, triggering an interferon response and causing Aicardi—Goutières Syndrome [122,123].

AGS is a severe Mendelian inflammatory disorder that affects particularly the brain and frequently causes death in childhood. The disease is characterized by progressive encephalopathy, psychomotor regression, and lesions of the skin, together with increased levels of Type I IFN in the cerebrospinal fluid and serum, and induction of ISGs detectable in peripheral blood. AGS is associated with mutations in seven genes, all involved with nucleic acid metabolism or signaling: TREX1, RNASEH2 A/B/C, SAMHD1, ADAR1 and IFIH1 [124–126]. Most of these genes also have been linked with the innate immune system that restricts retroviral infection and suppresses endogenous retrotransposons [23,123,127,128]. We predict mutations in additional anti-retroelement genes, perhaps even including ZAP or MOV10, will be linked to inflammatory diseases involving interferon overexpression.

## Materials and Methods

### Plasmid and RNAi constructs

Constructs CEP-EGFP, pc-L1-1FH, pc-L1-RP, ORF1-GFP L1-RP, 99-PUR-L1-RP-MS2-6X, pCEP-5′UTR-ORF2-No-Neo, and XRN1-RFP have been described [63,64]. ZAP-L-FL with a C-terminal FLAG-tag, and HA-ZAP 1–256 and HA-ZAP 253–902 with N-terminal HA-tags were generated by PCR and cloned in pcDNA6 myc/hisB vector. Ultimate ORF cDNA clones (Invitrogen) were cloned with V5-epitope tags and tobacco etch virus (TEV) protease cleavage sites on their N-termini by shuttling them from pENTR221 vector into pcDNA3.1/nV5-DEST vector using Gateway Technology (Invitrogen). Ultimate ORF Clone ID numbers are shown in S1 Table.

Clones obtained as gifts included Alu-neo^Tet^ and IAP-neo^TNF^ (M. Dewannieux, Institut Gustave Roussy, Villejuif [61,62]), DHX30(v2)-HA and DHX30(v2)-RFP (C. Liang, Lady Davis Institute-Jewish General Hospital, Montreal [106]), pcDNA3.1-V5-His-MOV10 (Y.-H. Zheng, Michigan State University, East Lansing [129]), ORFeus-Mm (WA-125) (W. An, Washington State Univ. [130]), ORFeus-HS (WA117) (L. Dai, Johns Hopkins School of Medicine, Baltimore [57]), pCDNA3.1-V5-His full-length PARP1 (J. Pascal, Thomas Jefferson Univ., Philadelphia [131]), GFP-PARP13.2 (GFP-ZAP-S; A Leung, Johns Hopkins School of Medicine [89]), SBP-PARP13.1, SBP-PARP13.1^ΔZnF^ and SBP-PARP13.1^VYFHR^ (P Chang, MIT, Cambridge [54]), pDest51-USP9X-V5 (R. Hughes, Buck Institute for Research on Aging, Novato [132]), Myc-USP7 (Y. Sheng, York University, Toronto [133]), pcDNA-Vphu (a codon-optimized version of the native Vpu gene; NIH AIDS Reagent Program [40]), pCEP-5′UTR-ORF2-No-Neo (J.L. García-Pérez, GENYO, Spain) [134]), and HA-ZAP-L, HA-ZAP-S, and Rat HA-NZAP (H. Malik, Fred Hutchinson Cancer Research Center, Seattle [32]). An altered amino acid (M201K) was restored to consensus in HA-ZAP-L, and the change was found to have no effect on L1 retrotransposition. pEasiLV-MCS MX2-Flag WT was obtained from M. Malim (King's College, London [45]) and the MX2 gene was amplified with C-terminal V5-tag by PCR and recloned in the vector pcDNA3. The K131A mutant was generated by the Quikchange Site-Directed Mutagenesis method (Agilent Technologies).

siRNAs were generated by Sigma-Aldrich based on the following sense sequences:

siCNT3 AUGUAUUGGCCUGUAUUAG[dT][dT],

siZC3HAV1-2 1435–1453 UUGGGUCAGCAUCAUCUGC[dT][dT],

siZC3HAV1-3 1637–1655 AUGUGCUCAAAGUCCGUCC[dT][dT] [60], and

siCNT4 UAAGGCUAUGAAGAGAUAC[dT][dT].

### Immunoprecipitation and MS sequencing

For MS sequence determination, HEK 293T cells were transfected in T_75_ flasks with 15 μg of ZAP-L-FL or pcDNA6 myc/hisB (Invitrogen) empty vector and expanded for approximately 45 hr, followed by whole cell lysate preparation by sonication. IP and sample recovery were as previously described [46]. Treatment of samples with 30 μg/ml DNase-free RNase (Roche) was in the absence of RNase inhibitors. MS sequencing and database analyses was performed by the Johns Hopkins Mass Spectrometry and Proteomics Facility as previously described [46].

For each co-IP, extracts from approximately 6×10^6^ 293T cells in T_75_ flasks transfected with ZAP-L-FL and test protein constructs were prepared in 750 μl of lysis buffer supplemented with protease, phosphatase, and RNase inhibitors, and immunoprecipitated as previously described [46]. Lysates containing test proteins of predominantly nuclear localization were sonicated. RNase-treated reactions contained 25 μg/ml RNase, DNase-free HC (Roche) and 25 μg/ml RNaseA (Invitrogen) and no RNase inhibitors.

### Cell culture and retrotransposition assay

Human 2102Ep embryonal carcinoma cells (a gift from P. K. Andrews, University of Sheffield), HeLa-HA and HeLa-JVM cells ([135]; gifts from J.L. García-Pérez, GENYO, Spain), and HEK 293T cells (ATCC) were grown in Dulbecco’s modified Eagle’s medium with 10% FBS (Hyclone), GlutaMax and Pen-Strep (Invitrogen). Plasmid and siRNA transfections used FuGENE HD (Promega) and Lipofectamine RNAiMAX (Life Technologies) reagents, respectively.

The EGFP L1 cell culture retrotransposition assay was conducted as previously described [27,38]. 2.5×10^5^ HeLa or 293T cells/well were seeded in 6-well dishes. The following day, 1.0 μg of 99-PUR-RPS-EGFP, a plasmid containing L1-RP and the EGFP retrotransposition reporter cassette, was cotransfected with 0.5 μg of empty vector (pcDNA3 or pcDNA6 myc/hisB, Invitrogen) or test plasmid. All transfections were in quadruplicate wells. Five days post-transfection, cells having a retrotransposition event, and hence expressing EGFP, were assayed by flow cytometry. Gating exclusions were based on background fluorescence of plasmid 99-PUR-JM111-EGFP, an L1 construct containing two point mutations in ORF1 that abolish retrotransposition [37]. Within each experiment, results were normalized to fluorescence of 99-PUR-RPS-EGFP cotransfected with empty vector.

The Alu retrotransposition assay was carried out essentially as described in Dewannieux et al. [61]. Retrotransposition construct Alu-neo^Tet^ was cotransfected in HeLa-HA cells with pcDNA6 myc/hisB empty vector or retrotransposition driver plasmid pCEP-5′UTR-ORF2-No-Neo, together with test plasmids. Eighteen hours post-transfection, HeLa-HA cells were expanded from six-well plates to T_75_ flasks, and three days later selection for retrotransposition events with 550 μg/ml of G418 was begun. After 15 days of selection, cells were fixed, stained with Giemsa, and colonies were counted.

Similarly, 1.0 μg of the IAP element reporter plasmid, IAP-neo^TNF^ [62], was cotransfected with 0.5 μg empty vector or test plasmid in HeLa-JVM cells, selected with G418, and colony numbers were counted.

### Assessment of toxicity

To reveal any differences in transfection efficiencies of test proteins or off-target effects on EGFP reporter expression, we followed the strategy of Wei et al. (39). Each test plasmid (0.5 μg) was co-transfected in quadruplicate wells of 12-well plates with CEP-EGFP (0.5 μg), a construct that constitutively expresses EGFP from a CMV promoter. Four days post-transfection, EGFP fluorescence was determined by flow cytometry, as previously described [46].

To determine potential cell toxicity caused by test proteins, 18,000 293T cells were seeded in 75 μl of Dulbecco’s modified Eagle’s complete medium in 96-well plates. The next day, transfection reactions prepared with 70 ng of test plasmid, 0.2 μl of Fugene HD and 25 μl of Opti-MEM Reduced Serum Medium (Invitrogen) were added to each well. After 3 or 4 days, a MultiTox-Fluor Multiplex Cytotoxicity Assay kit (Promega) was used to assay cell toxicity, as previously described [46].

To further test potential toxicity from expression of ZAP, we co-transfected in HeLa cells pcDNA6 myc/his B, a *bsr* expression vector, together with either empty vector (pcDNA3) or ZAP expression constructs. On day 2, cells were expanded to T_75_ flasks and selection with 2 μg/ml blasticidin was begun. After 12 days, cells were fixed, stained and colonies were counted. Cytotoxicity will reduce total colony counts compared with empty vector control (S2B Fig).

### Immunofluorescence and western blotting

Commercial antibodies included mouse (ms) α-V5-tag (Invitrogen), ms α-FLAG-tag (Sigma), rabbit (rb) α-HA-tag (C29F4), rb α-HSP90 and rb α-Myc-tag (71D10)(Cell Signaling Technology), goat (gt) α- anti-eIF3η (N-20), gt α-MX2 (C-20), ms α-SBP (SB19-C4), and gt α-TIA1 (C-20)(Santa Cruz Biotechnology), rb α-β-tubulin-2 (Pierce), and rb α-MOV10 and rb α-ZC3HAV1 (ProteinTech). Donkey Cy3-, Cy5-, DyLight 488-, or DyLight 549-conjugated, and HRP-conjugated secondary antibodies were from Jackson ImmunoResearch Laboratories. Purified polyclonal α-ORF1p (AH40.1) and monoclonal α-ORF1p (α-moORF1) antibodies were gifts from M. Singer (Carnegie Institution of Washington [136]) and K. Burns (Johns Hopkins School of Medicine [137]), respectively.

Western blotting, IF, and FISH were performed as described [63,64].

### Reverse transcriptase and RNA analyses

L1 ORF2p reverse transcriptase analysis followed the LEAP protocol [81]. Primers used were:

3′RACE adapter NV: GCGAGCACAGAATTAATACGACTCACTATAGGTTTTTTTTTTTTVN

3′RACE outer: GCGAGCACAGAATTAATACGACT

bORF2-end2, GATGAGTTCATATCCTTTGTAGGG

The sequence of the antisense RNA-FISH probe Cy2-MS2 was,

Cy3-GTCGACCTGCAGACATGGGTGATCCTCATGTTTTCTAGGCAATTA.

Cells were lysed and their RNA initially extracted with Trizol (Life Technologies), followed by further purification using an RNeasy Mini Kit (Qiagen). Residual DNA was removed by Turbo DNA-free Kit DNase treatment (Ambion), and cDNA was generated from the RNA using the SuperScript III First Strand Synthesis System (Invitrogen) and a polyT primer. Subsequent PCR used GoTaq DNA polymerase (Promega). RT-PCR primers were:

1EGFPcass5P TGTTCTGCTGGTAGTGGTCG

2EGFPcass3P TATATCATGGCCGACAAGCAG,

which span the intron of the 99-PUR-JM111-EGFP reporter cassette, and

13HSPA6for CAAAATGCAAGACAAGTGTCG

14HSPA6rev TTCTAGCTTTGGAGGGAAAG,

which amplify HSPA6 (Accession No. NM_002155).

## Supporting Information

S1 Fig Endogenous MX2 protein is induced by interferon in HeLa-JVM cells only. Cells were treated with 1000 U/ml Universal Type I Interferon Alpha (PML Assay Science) or left untreated for 48 hours. Plasmid-expressed MX2-V5 is shown in lane 7.
(TIF)

S2 Fig ZAP isoforms restrict variant L1 constructs in multiple cell types without obvious cell toxicity or plasmid degradation. (A) Similar to 293T cells, ectopic expression of ZAP restricts retrotransposition of 99-PUR-RPS-EGFP in HeLa-JVM cells. (B) Overexpressed ZAP constructs have minimal effect on cell viability. HeLa-JVM cells were cotransfected with 0.05 μg of pcDNA6 myc/hisB and 0.5 μg of plasmid containing ZAP or unrelated proteins (C22ORF28 or KIF11) in six-well plates, and after two days were expanded to T_75_ flasks. Cells were then selected for two weeks with 2 μg/ml blasticidin, fixed, and stained with giemsa. Colony counts are normalized to empty vector transfection. Standard deviation is for three separate flasks in a single experiment. (C) Expression of ZAP constructs restrict cell culture retrotransposition of an EGFP reporter-tagged and codon-optimized human L1 (ORFeus-HS, WA117 [57]). Results for a single experiment are show (quadruplicate wells). (D) Similarly, ZAP constructs inhibit retrotransposition of ORFeus-Mm (WA125 [129]), a codon-optimized version of mouse L1_spa_ [59] cloned downstream of a CMV promoter. Results are for a single experiment. (E) Expression of ZAP does not affect the integrity of a contransfected L1 reporter construct. Vector alone, HA-ZAP-S or HA-ZAP-L was cotransfected with 99-PUR-JM111-EGFP, containing a mutant L1 defective for retrotransposition. At 3 days post-transfection, 293T cells were harvested, HIRT DNA extraction was performed, and plasmid DNA was analyzed using PCR and primers that flank the intron of the L1 EGFP reporter cassette.
(TIF)

S3 Fig Protein colocalization analyses of 293T cells. (A) ZAP-interacting helicase DHX30 tagged with RFP colocalizes with GFP-tagged ORF1p in cytoplasmic granules. (B) ZAP-L and ZAP-S isoforms colocalize in large cytoplasmic foci.
(TIF)

S4 Fig Reprobing of the western blots of Fig 4B with α-FLAG antibody. ZAP-L-FL protein immunoprecipates efficiently in the presence of test proteins and RNase.
(TIF)

S5 Fig V5-TEV-MX1 and MX2-V5 co-IP with ZAP-L-FL from 293T cells. The interactions are resistant to digestion by RNase. V5-TEV-ISG20 also weakly interacts with ZAP-L-FL in the absence of RNase. IP conditions were as described for Fig 4B.
(TIF)

S1 Table Detailed summary of MS analyses of proteins that co-IP with FLAG-tagged ZAP complexes from 293T cells. (PDF)
