# Supplementary figures and images for: The Broad-Spectrum Antiviral Protein ZAP Restricts Human Retrotransposition
Source: PLoS Genet. 2015 May 22;11(5):e1005252. doi: 10.1371/journal.pgen.1005252 (PMC4441479; doi:10.1371/journal.pgen.1005252)

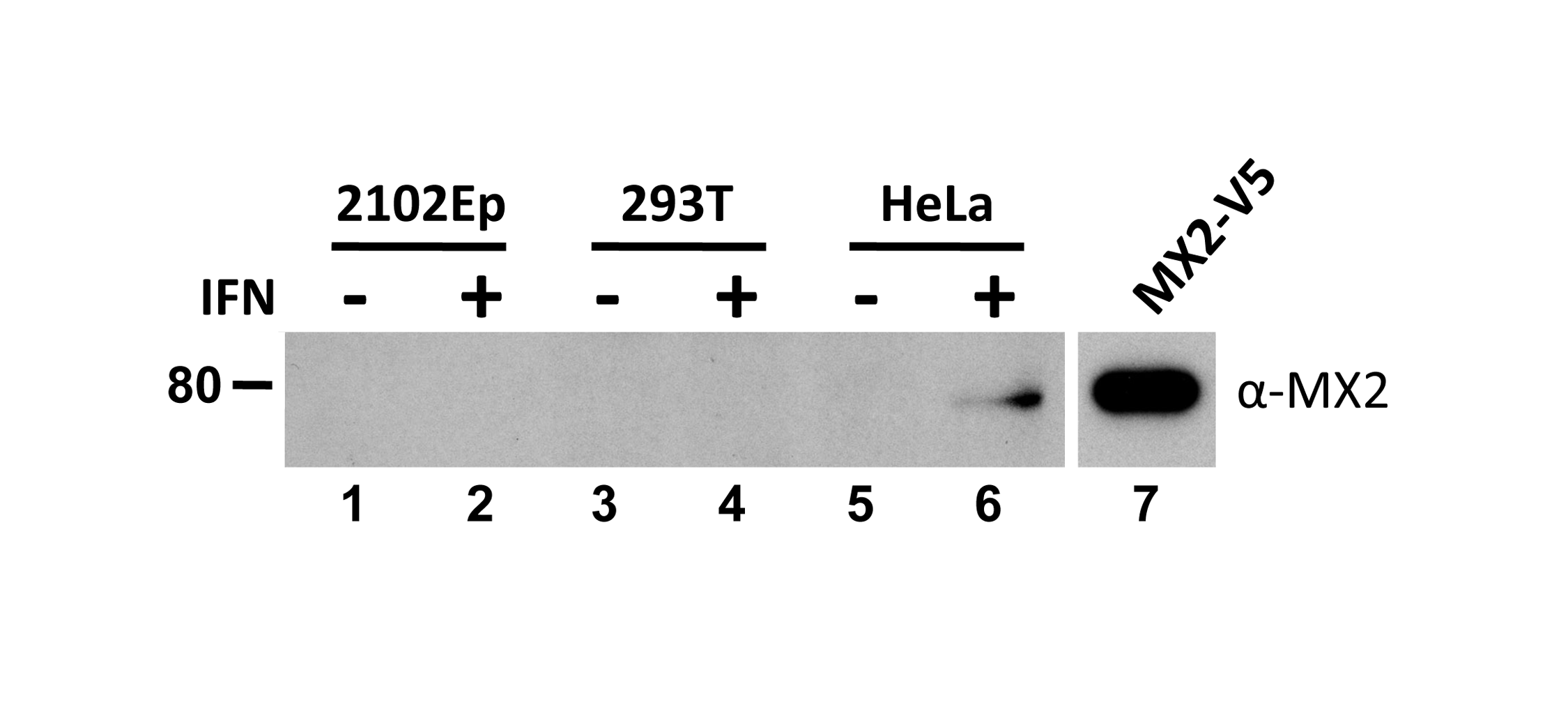

Supplement: S1 Fig — Cells were treated with 1000 U/ml Universal Type I Interferon Alpha (PML Assay Science) or left untreated for 48 hours. Plasmid-expressed MX2-V5 is shown in lane 7. (TIF) [file pgen.1005252.s001.tif]

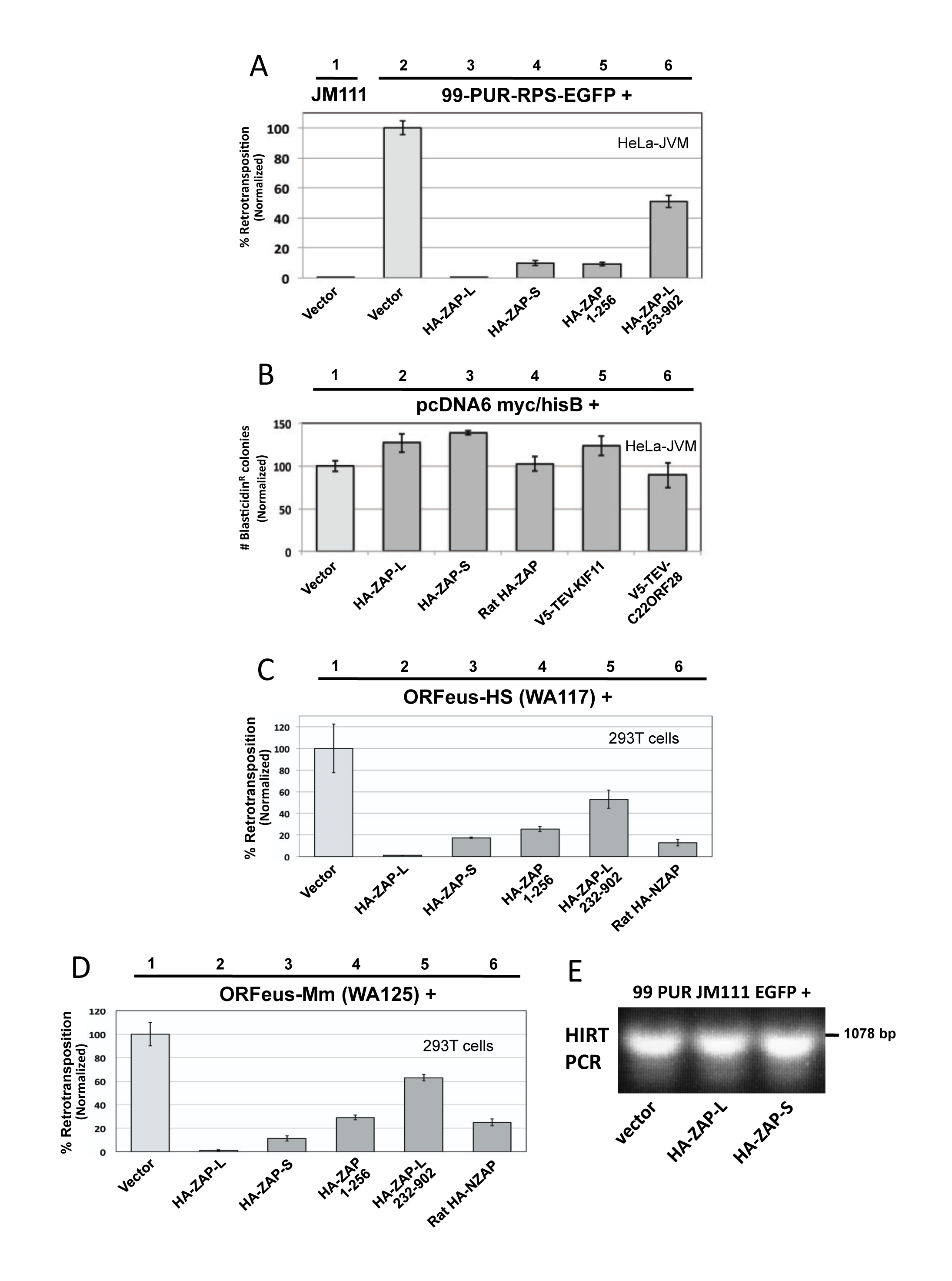

Supplement: S2 Fig — (A) Similar to 293T cells, ectopic expression of ZAP restricts retrotransposition of 99-PUR-RPS-EGFP in HeLa-JVM cells. (B) Overexpressed ZAP constructs have minimal effect on cell viability. HeLa-JVM cells were cotransfected with 0.05 μg of pcDNA6 myc/hisB and 0.5 μg of plasmid containing ZAP or unrelated proteins (C22ORF28 or KIF11) in six-well plates, and after two days were expanded to T75 flasks. Cells were then selected for two weeks with 2 μg/ml blasticidin, fixed, and stained with giemsa. Colony counts are normalized to empty vector transfection. Standard deviation is for three separate flasks in a single experiment. (C) Expression of ZAP constructs restrict cell culture retrotransposition of an EGFP reporter-tagged and codon-optimized human L1 (ORFeus-HS, WA117 [57]). Results for a single experiment are show (quadruplicate wells). (D) Similarly, ZAP constructs inhibit retrotransposition of ORFeus-Mm (WA125 [129]), a codon-optimized version of mouse L1spa [59] cloned downstream of a CMV promoter. Results are for a single experiment. (E) Expression of ZAP does not affect the integrity of a contransfected L1 reporter construct. Vector alone, HA-ZAP-S or HA-ZAP-L was cotransfected with 99-PUR-JM111-EGFP, containing a mutant L1 defective for retrotransposition. At 3 days post-transfection, 293T cells were harvested, HIRT DNA extraction was performed, and plasmid DNA was analyzed using PCR and primers that flank the intron of the L1 EGFP reporter cassette. (TIF) [file pgen.1005252.s002.tif]

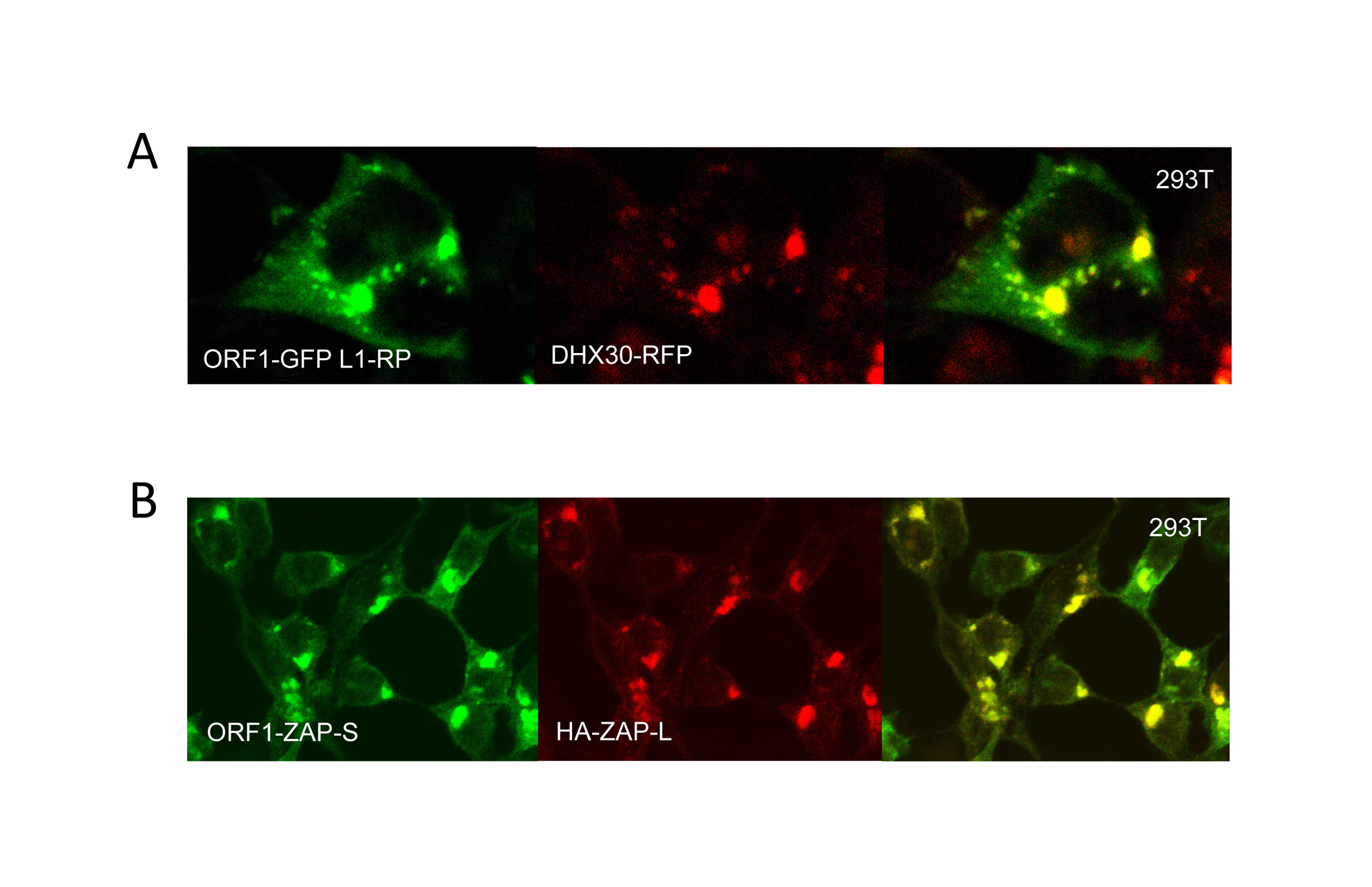

Supplement: S3 Fig — (A) ZAP-interacting helicase DHX30 tagged with RFP colocalizes with GFP-tagged ORF1p in cytoplasmic granules. (B) ZAP-L and ZAP-S isoforms colocalize in large cytoplasmic foci. (TIF) [file pgen.1005252.s003.tif]

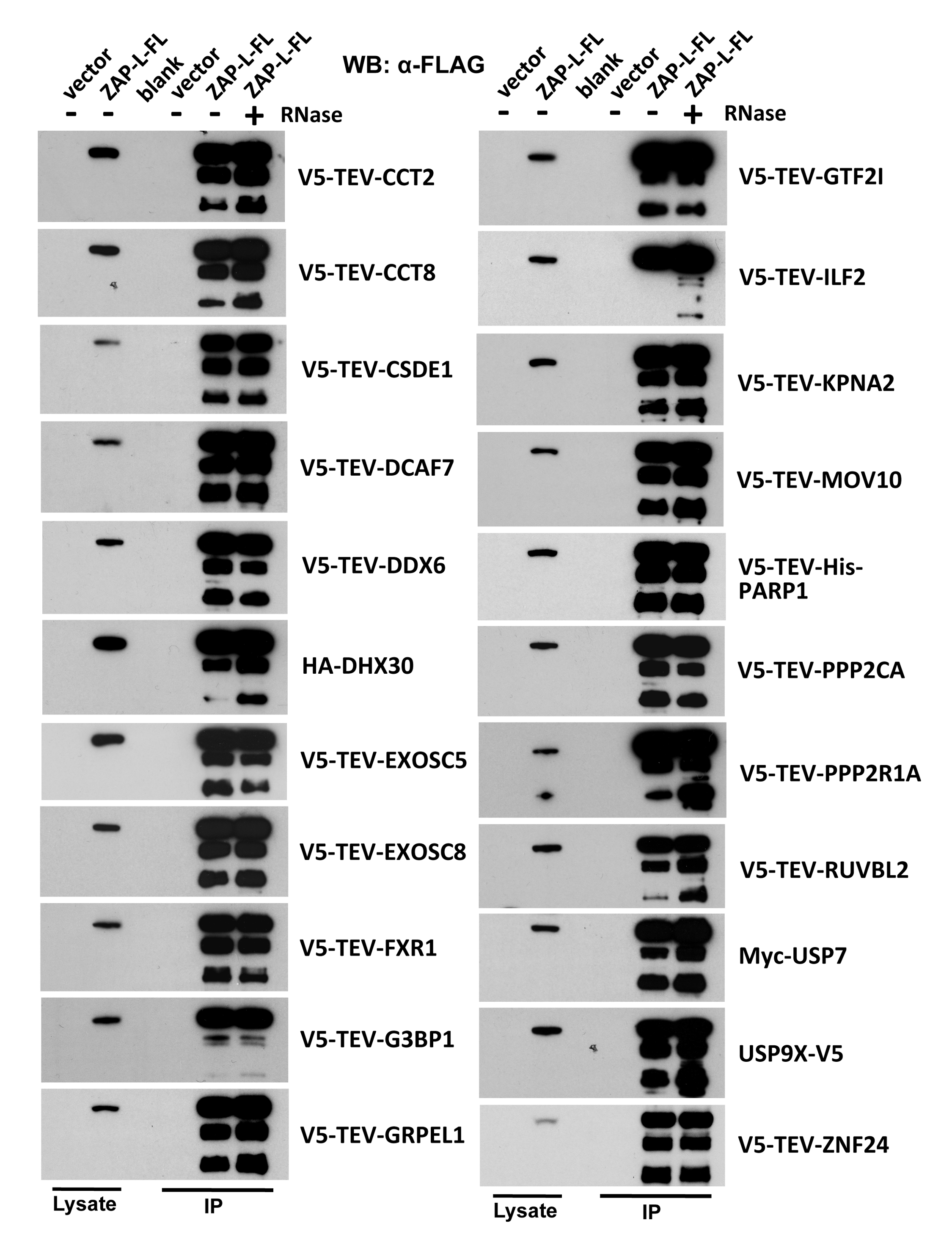

Supplement: S4 Fig — ZAP-L-FL protein immunoprecipates efficiently in the presence of test proteins and RNase. (TIF) [file pgen.1005252.s004.tif]

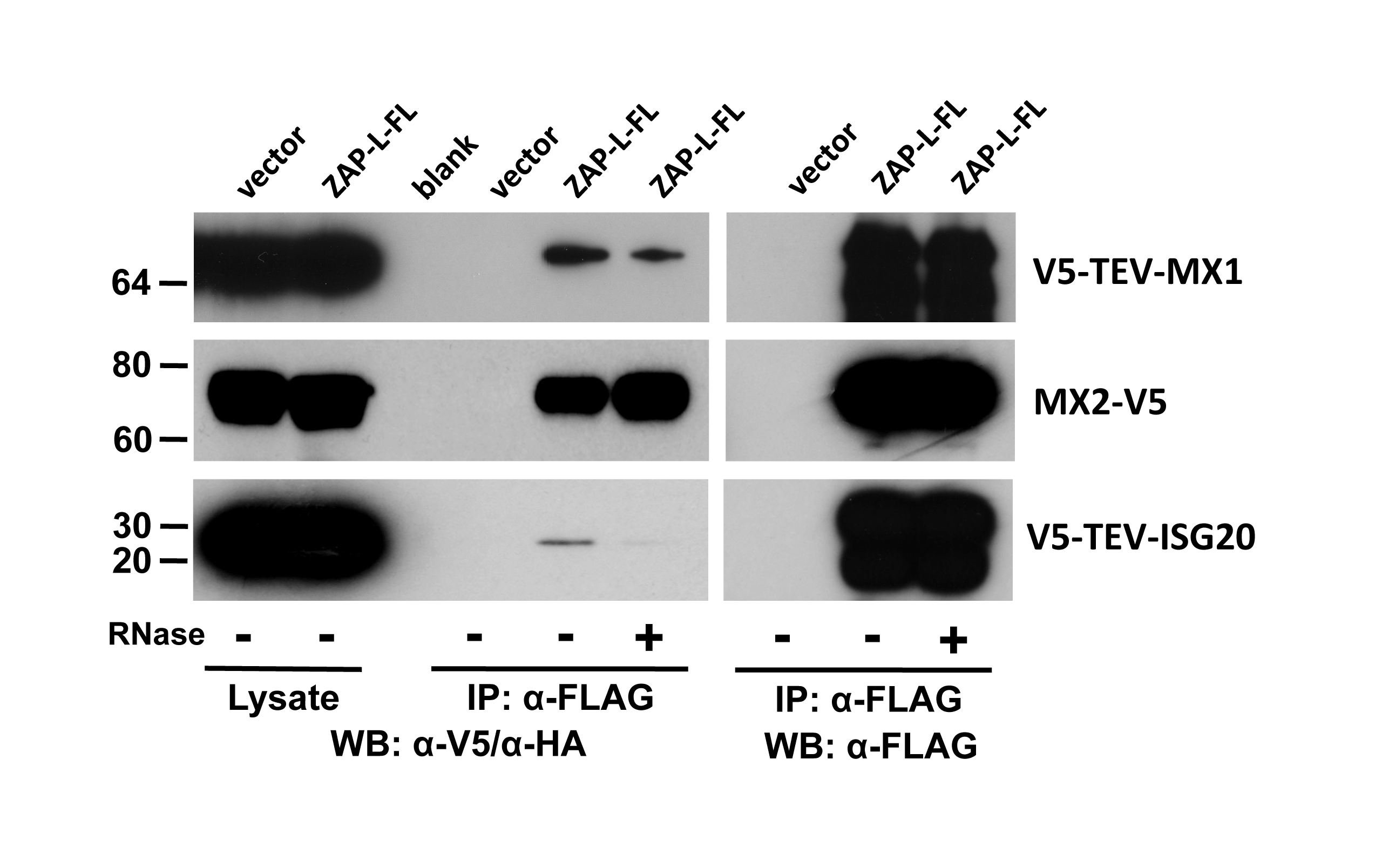

Supplement: S5 Fig — The interactions are resistant to digestion by RNase. V5-TEV-ISG20 also weakly interacts with ZAP-L-FL in the absence of RNase. IP conditions were as described for Fig 4B. (TIF) [file pgen.1005252.s005.tif]
