# Supplementary material for: The Broad-Spectrum Antiviral Protein ZAP Restricts Human Retrotransposition
Source: PLoS Genet. 2015 May 22;11(5):e1005252. doi: 10.1371/journal.pgen.1005252 (PMC4441479; doi:10.1371/journal.pgen.1005252)
Supplement: S1 Table — (PDF) [file pgen.1005252.s006.pdf]

**S1 Table. Detailed summary of MS analyses of proteins that co-IP with FLAG-tagged ZAP complexes from 293T cells.**

| | Protein Name | Gene Symbol | Alternate Gene Symbol | In Interferome Database (hs: human/mm: mouse) | Accession Number | Ultimate ORF Clone # | Molecular wt (kD) | # Unique Peptides | | # Assigned Spectra | | # Unique Spectra | | Percentage of Total Spectra | |
|---|---|---|---|---|---|---|---|---|---|---|---|---|---|---|---|
| | | | | | | | | RNase | | | | | | | |
| | | | | | | | | - | + | - | + | - | + | - | + |
| 1 | Apoptosis-inducing factor, mitochondrion-associated, 1 | AIFM1 | | | NM_001130846 | | 66 | 5 | 4 | 5 | 4 | 5 | 4 | 0.06% | 0.05% |
| 2 | A kinase (PRKA) anchor protein 8 | AKAP8 | | mm | NM_014371 | | 72 | 2 | 3 | 3 | 4 | 3 | 4 | 0.03% | 0.05% |
| 3 | Carbamoyl-phosphate synthetase 2, aspartate transcarbamylase, and dihydroorotase | CAD | | | NM_004341 | | 243 | 11 | 31 | 13 | 37 | 12 | 35 | 0.15% | 0.41% |
| 4 | Cell cycle associated protein 1 | CAPRIN1 | | | NM_005898 | | 78 | 4 | 0 | 4 | 0 | 4 | 0 | 0.05% | 0 |
| 5 | Coiled-coil domain containing 86 | CCDC86 | Cyclon | mm | NM_024098 | | 40 | 3 | 0 | 3 | 0 | 3 | 0 | 0.03% | 0 |
| 6 | T-complex protein 1, alpha subunit | CCT1 | TCP-1α | | NM_030752 | | 60 | 9 | 6 | 12 | 7 | 12 | 7 | 0.14% | 0.08% |
| 7 | Chaperonin containing TCP1, subunit 2 (beta) | CCT2 | TCP-1β | | NM_001198842 | IOH41691 | 57 | 17 | 16 | 62 | 52 | 32 | 28 | 0.28% | 0.24% |
| 8 | Chaperonin containing TCP1, subunit 3 (gamma) | CCT3 | TCP-1γ | mm | NM_005998 | | 61 | 9 | 6 | 9 | 6 | 9 | 6 | 0.10% | 0.07% |
| 9 | Chaperonin containing TCP1, subunit 4 (delta) | CCT4 | TCP-1δ | mm | NM_006430 | | 58 | 7 | 7 | 20 | 19 | 11 | 11 | 0.09% | 0.09% |
| 10 | Chaperonin containing TCP1, subunit 6A (zeta 1) | CCT6A | TCP-1ζ | | NM_001762 | | 58 | 10 | 6 | 14 | 8 | 12 | 7 | 0.16% | 0.09% |
| 11 | Chaperonin containing TCP1, subunit 7 (eta) | CCT7 | TCP-1η | mm | NM_006429 | | 59 | 6 | 8 | 17 | 26 | 10 | 16 | 0.08% | 0.12% |
| 12 | Chromodomain helicase DNA binding protein 4 | CHD4 | | mm | NM_001273 | | 218 | 6 | 7 | 7 | 8 | 7 | 8 | 0.08% | 0.09% |
| 13 | Cold shock domain containing E1, RNA-binding | CSDE1 | | hs | NM_001130523 | IOH21647 | 91 | 3 | 0 | 3 | 2 | 3 | 2 | 0.03% | 0.02% |
| 14 | DDB1 and CUL4 associated factor 7 | DCAF7 | | | NM_005828 | IOH3135 | 39 | 0 | 3 | 0 | 3 | 0 | 3 | 0 | 0.02% |
| 15 | DEAD (Asp-Glu-Ala-Asp) box polypeptide 18 | DDX18 | | | NM_006773 | | 75 | 3 | 0 | 3 | 0 | 3 | 0 | 0.03% | 0 |
| 16 | DEAD (Asp-Glu-Ala-Asp) box polypeptide 6 | DDX6 | RCK, P54 | mm | NM_004397 | IOH61588 | 54 | 5 | 0 | 5 | 0 | 5 | 0 | 0.06% | 0 |
| 17 | DEAH (Asp-Glu-Ala-His) box polypeptide 30 | DHX30 | DDX30 | | NM_014966 | | 129 | 23 | 6 | 25 | 7 | 25 | 7 | 0.28% | 0.08% |
| 18 | DEAH (Asp-Glu-Ala-Asp/His) box polypeptide 57 | DHX57 | | hs | NM_198963 | | 156 | 4 | 0 | 4 | 0 | 4 | 0 | 0.05% | 0 |
| 19 | DnaJ (Hsp40) homolog, subfamily A, member 1 | DNAJA1 | | hs | NM_001539 | | 45 | 5 | 7 | 5 | 7 | 5 | 7 | 0.06% | 0.08% |
| 20 | DnaJ (Hsp40) homolog, subfamily A, member 2 | DNAJA2 | | | NM_005880 | | 46 | 1 | 4 | 1 | 4 | 1 | 4 | 0.01% | 0.05% |
| 21 | ELAV (embryonic lethal, abnormal vision, Drosophila)-like 1 (Hu antigen R) | ELAVL1 | HUR | | NM_001419 | IOH2942 | 36 | 3 | 1 | 4 | 2 | 3 | 1 | 0.05% | 0.02% |
| 22 | Glutamyl-prolyl-tRNA synthetase | EPRS | | | NM_004446 | | 171 | 7 | 9 | 7 | 10 | 7 | 10 | 0.08% | 0.11% |
| 23 | Endoplasmic reticulum protein 44 | ERP44 | | hs | NM_015051 | | 47 | 15 | 18 | 22 | 26 | 19 | 24 | 0.25% | 0.29% |
| 24 | Exosome component 8 | EXOSC8 | RRP43 | mm | NM_181503 | IOH10049 | 30 | 2 | 2 | 6 | 6 | 4 | 4 | 0.03% | 0.03% |
| 25 | FtsJ homolog 3 (E. coli) | FTSJ3 | | | NM_017647 | | 97 | 4 | 0 | 4 | 1 | 4 | 1 | 0.05% | 0.01% |
| 26 | Fragile X mental retardation, autosomal homolog 1 | FXR1 | | | NM_005087 | IOH22379 | 70 | 7 | 7 | 7 | 7 | 7 | 7 | 0.08% | 0.08% |
| 27 | Fragile X mental retardation, autosomal homolog 2 | FXR2 | | hs | NM_004860 | | 74 | 1 | 3 | 1 | 3 | 1 | 3 | 0.01% | 0.03% |
| 28 | GTPase activating protein (SH3 domain) binding protein 1 | G3BP1 | | mm | NM_198395 | IOH7337 | 52 | 3 | 1 | 3 | 1 | 3 | 1 | 0.03% | 0.01% |
| 29 | GTPase activating protein (SH3 domain) binding protein 2 | G3BP2 | | hs | NM_203504 | | 51 | 3 | 0 | 15 | 0 | 6 | 0 | 0.07% | 0 |
| 30 | Guanine nucleotide binding protein-like 3 (nucleolar) /nucleostemin | GNL3 | | | NM_014366 | | 62 | 9 | 2 | 11 | 2 | 11 | 2 | 0.12% | 0.02% |
| 31 | GrpE-like 1, mitochondrial (E. coli) | GRPEL1 | | | NM_025196 | IOH10877 | 24 | 10 | 11 | 14 | 16 | 12 | 16 | 0.16% | 0.18% |
| 32 | General transcription factor IIi | GTF2I | | hs | NM_032999 | | 112 | 8 | 15 | 8 | 18 | 8 | 18 | 0.09% | 0.20% |
| 33 | General transcription factor IIIC, polypeptide 1, alpha 220kDa | GTF3C1 | | | NM_001520 | | 239 | 0 | 3 | 0 | 3 | 0 | 3 | 0 | 0.03% |
| 34 | General transcription factor IIIC, polypeptide 3, 102kDa | GTF3C3 | TFIIIC102 | | NM_012086 | | 101 | 0 | 3 | 1 | 3 | 1 | 3 | 0.01% | 0.03% |
| 35 | GTP binding protein 4 | GTPBP4 | | mm | NM_012341 | | 74 | 9 | 0 | 11 | 0 | 10 | 0 | 0.12% | 0 |
| 36 | Histone deacetylase 1 | HDAC1 | | | NM_004964 | | 55 | 3 | 2 | 3 | 2 | 3 | 2 | 0.03% | 0.02% |
| 37 | Heterochromatin protein 1-binding protein 3 | HP1BP3 | | | NM_016287 | | | 3 | 0 | 3 | 0 | 3 | 0 | 0.03% | 0.00% |
| 38 | Heterogeneous nuclear ribonucleoprotein A0 | HNRNPA0 | | hs | NM_006805 | | 31 | 4 | 0 | 4 | 0 | 4 | 0 | 0.05% | 0 |
| 39 | heterogeneous nuclear ribonucleoprotein U-like 1 | HNRPUL1 | | | NM_007040 | | 85 | 6 | 0 | 6 | 0 | 6 | 0 | 0.07% | 0 |
| 40 | Heat shock 70kDa protein 4 | HSPA4 | HSP70 | hs/mm | NM_002154 | | 94 | 1 | 7 | 1 | 7 | 1 | 7 | 0.01% | 0.08% |
| 41 | Heat shock 105kDa/110kDa protein 1 | HSPH1 | | | NM_006644 | | 97 | 2 | 4 | 2 | 4 | 2 | 4 | 0.02% | 0.05% |
| 42 | Insulin-like growth factor 2 mRNA binding protein 3 | IGF2BP3 | IMP3 | hs | NM_006547 | | 64 | 8 | 0 | 8 | 0 | 8 | 0 | 0.09% | 0 |
| 43 | Interleukin enhancer-binding factor 2 | ILF2 | | | NM_004515 | IOH3455 | 43 | 11 | 7 | 16 | 7 | 14 | 7 | 0.18% | 0.08% |
| 44 | Karyopherin alpha 2 (RAG cohort 1, importin alpha 1) | KPNA2 | | mm | NM_002266 | IOH28926 | 58 | 3 | 3 | 3 | 3 | 3 | 3 | 0.03% | 0.03% |
| 45 | KRI1 homolog (S. cerevisiae) | KRI1 | | | NM_023008 | | 83 | 7 | 0 | 7 | 0 | 7 | 0 | 0.08% | 0 |
| 46 | La ribonucleoprotein domain family, member 1 | LARP1 | | | NM_015315 | | 116 | 5 | 0 | 5 | 0 | 5 | 0 | 0.06% | 0 |

| | | | | | | | | | | | | | | | |
|---|---|---|---|---|---|---|---|---|---|---|---|---|---|---|---|
| 47 | La ribonucleoprotein domain family, member 7 | LARP7 | | | NM_015454 | | 67 | 4 | 0 | 4 | 0 | 4 | 0 | 0.05% | 0 |
| 48 | Ly1 antibody reactive | LYAR | | | NM_017816 | | 44 | 6 | 0 | 3 | 0 | 3 | 0 | 0.03% | 0 |
| 49 | Moloney leukemia virus 10, homolog (mouse) | MOV10 | | hs/mm | NM_020963 | IOH4005 | 114 | 3 | 1 | 3 | 1 | 3 | 1 | 0.03% | 0.01% |
| 50 | MYB binding protein (P160) 1a | MYBBP1A | | mm | NM_014520 | | 149 | 4 | 3 | 4 | 3 | 4 | 3 | 0.05% | 0.03% |
| 51 | NOP2 nucleolar protein | NOP2 | | | NM_001258308 | | 89 | 6 | 0 | 7 | 0 | 7 | 0 | 0.08% | 0 |
| 52 | poly [ADP-ribose] polymerase 1 [Homo sapiens] | PARP1 | | mm | NM_001618 | | 113 | 0 | 3 | 0 | 3 | 0 | 3 | 0 | 0.03% |
| 53 | Protein phosphatase 2, catalytic subunit, alpha isozyme | PPP2CA | | | NM_002715 | IOH5263 | 36 | 6 | 7 | 6 | 7 | 6 | 7 | 0.07% | 0.08% |
| 54 | Protein phosphatase 2, regulatory subunit A, alpha | PPP2R1A | PR65A | | NM_014225 | IOH13670 | 65 | 13 | 16 | 16 | 24 | 16 | 21 | 0.18% | 0.27% |
| 55 | Protein phosphatase 2, regulatory subunit A, beta | PPP2R1B | | | NM_001177562 | | 53 | 9 | 10 | 10 | 13 | 10 | 13 | 0.11% | 0.14% |
| 56 | Pentatricopeptide repeat domain 3 | PTCD3 | | | NM_017952 | | 79 | 11 | 11 | 16 | 13 | 15 | 13 | 0.18% | 0.14% |
| 57 | Pyrroline-5-carboxylate reductase-like | PYCRL | | | NM_023078 | | 30 | 0 | 3 | 1 | 3 | 1 | 3 | 0.01% | 0.03% |
| 58 | RNA binding motif protein 14 | RBM14 | | | NM_006328 | | 69 | 0 | 3 | 1 | 3 | 1 | 3 | 0.01% | 0.03% |
| 59 | Reticulocalbin 2, EF-hand calcium binding domain | RCN2 | | | NM_002902 | | 37 | 5 | 4 | 5 | 4 | 5 | 4 | 0.06% | 0.05% |
| 60 | Ribonuclease/angiogenin inhibitor 1 | RNH1 | | hs | NM_002939 | | 50 | 6 | 0 | 8 | 0 | 6 | 0 | 0.09% | 0 |
| 61 | Ribosomal RNA processing 1B | RRP1B | | hs/mm | NM_015056 | | 84 | 4 | 0 | 5 | 0 | 4 | 0 | 0.06% | 0 |
| 62 | Ribosomal L1 domain containing 1 | RSL1D1 | CATX-11 | hs | NM_015659 | | 55 | 19 | 0 | 24 | 0 | 21 | 0 | 0.27% | 0 |
| 63 | RuvB-like 1 (E. coli) | RUVBL1 | TIP49A, PONTIN | mm | NM_003707 | | 50 | 2 | 3 | 2 | 3 | 2 | 3 | 0.02% | 0.03% |
| 64 | RuvB-like 2 (E. coli) | RUVBL2 | TIP49B, REPTIN | | NM_006666 | IOH3426 | 51 | 2 | 3 | 2 | 3 | 2 | 3 | 0.02% | 0.03% |
| 65 | Sjogren syndrome antigen B (autoantigen La) | SSB | LA | hs | NM_003142 | | 47 | 20 | 0 | 31 | 0 | 27 | 0 | 0.35% | 0 |
| 66 | Staufen double-stranded RNA binding protein 1 | STAU1 | | | NM_004602 | | 55 | 3 | 0 | 3 | 1 | 3 | 1 | 0.03% | 0.01% |
| 67 | Topoisomerase (DNA) I | TOP1 | | hs | NM_003286 | | 91 | 3 | 0 | 4 | 0 | 3 | 0 | 0.05% | 0 |
| 68 | Tripartite motif containing 25/E3 ubiquitin/ISG15 ligase TRIM25 | TRIM25 | | hs/mm | NM_005082 | IOH11168 | 71 | 6 | 2 | 7 | 2 | 7 | 2 | 0.08% | 0.02% |
| 69 | Tu translation elongation factor, mitochondrial | TUFM | | | NM_003321 | | 50 | 0 | 6 | 1 | 8 | 1 | 7 | 0.01% | 0.09% |
| 70 | ubiquitin specific peptidase 7 (herpes virus-associated) | USP7 | TIP48 | mm | NM_003470 | | 128 | 2 | 3 | 2 | 3 | 2 | 3 | 0.02% | 0.03% |
| 71 | Ubiquitin specific peptidase 9, X-linked | USP9X | FAF-X | | NM_001039590 | | 292 | 24 | 27 | 86 | 95 | 43 | 49 | 0.39% | 0.44% |
| 72 | 5'-3' exoribonuclease 2 | XRN2 | | mm | NM_012255 | | 109 | 12 | 6 | 12 | 6 | 12 | 6 | 0.14% | 0.07% |
| 73 | YTH domain containing 2 | YTHDC2 | | | NM_022828 | | 160 | 3 | 0 | 3 | 0 | 3 | 0 | 0.03% | 0 |
| 74 | YTH domain family, member 2 | YTHDF2 | | | NM_016258 | | 62 | 3 | 2 | 3 | 2 | 3 | 2 | 0.03% | 0.02% |
| 75 | Zinc finger CCCH-type, antiviral 1 | ZC3HAV1 | ZAP, PARP13 | hs | NM_020119 | IOH26215 | 101 | 72 | 84 | 666 | 890 | 132 | 155 | 7.50% | 9.90% |
| 76 | Zinc finger RNA binding protein | ZFR | | | NM_016107 | | 117 | 4 | 0 | 4 | 1 | 4 | 1 | 0.05% | 0.01% |
| 77 | Zinc finger protein 24 [Homo sapiens] | ZNF24 | | | NM_006965 | IOH4871 | 42 | 2 | 3 | 2 | 3 | 2 | 3 | 0.02% | 0.03% |
| 78 | Zinc finger protein 326 (ZNF326), transcript variant 1 | ZNF326 | | | NM_182976 | | 66 | 4 | 9 | 5 | 11 | 5 | 10 | 0.06% | 0.12% |
| | **Additional Ultimate ORF Clones (Invitrogen) used in this study.** | | | | | | | | | | | | | | |
| | Chaperonin containing TCP1, subunit 8 (theta) | CCT8 | | | NM_006585 | IOH40613 | | | | | | | | | |
| | Mitochondrial antiviral signaling protein | MAVS | VISA | | NM_001206491 | IOH26371 | | | | | | | | | |
| | Exosome component 5 | EXOSC5 | RRP46 | | NM_020158 | IOH6517 | | | | | | | | | |
| | Myxovirus (influenza virus) resistance 1, interferon-inducible protein p78 (mouse) | MX1 | MxA | | NM_002462 | IOH21990 | | | | | | | | | |
| | Radical S-adenosyl methionine domain containing 2 | RSAD2 | Viperin | | NM_080657 | IOH55127 | | | | | | | | | |
| | Bone marrow stromal cell antigen 2 | BST2 | Tetherin | | NM_004335 | IOH21990 | | | | | | | | | |
| | Interferon induced transmembrane protein 1 | IFITM1 | | | NM_003641 | IOH3214 | | | | | | | | | |
| | Tripartite motif containing 28 | TRIM28 | KAP1 | | NM_005762 | IOH5106 | | | | | | | | | |
| | Interferon stimulated exonuclease gene 20kDa | ISG20 | | | NM_002201 | IOH6793 | | | | | | | | | |
| | Chaperonin containing TCP1, subunit 6B (zeta 2) | CCT6B | | | NM_001193529 | IOH56061 | | | | | | | | | |
